# Chitosan activates a sequential Ca^**2+**^-ROS-NO-jasmonate signaling cascade governing withanolide accumulation in *Withania somnifera*

**DOI:** 10.1080/15592324.2026.2733235

**Published:** 2026-09-16

**Authors:** Shazia Perveen, Imran Haider, Aqib Mahmood, Sana ur Rehman, Muhammad Yousaf Nadeem, Azatullah Zaheer, Rashed N. Herqash, Omar M. Noman, Abdullah R. Alanzi

**Affiliations:** a Department of Zoology, The Women University Multan, Multan, Pakistan; b National Research Center of Intercropping, The Islamia University of Bahawalpur, Bahawalpur, Pakistan; c Department of Biology, Nakhchivan State University, Nakhchivan Azerbaijan; d College of Agriculture, Nanjing Agricultural University, Jiangsu, China; e Department of Business Administration, Faculty of Economics, Salam University, Kabul, Afghanistan; f Center for Research on Medicinal, Aromatic, and Poisonous Plants, Deanship of Scientific Research, King Saud University, Riyadh, Saudi Arabia

**Keywords:** *Withania somnifera*, chitosan elicitation, calcium signaling, NADPH oxidase, nitric oxide, jasmonate, withaferin A, untargeted metabolomics, molecular docking

## Abstract

Chitosan promotes withanolide accumulation in *W. somnifera; however,* the temporal organization of the underlying signaling network remains unresolved. We examined whether calcium (Ca^2^⁺), reactive oxygen species (ROS), nitric oxide (NO), and jasmone form an ordered cascade linking elicitor perception to specialized metabolism. Sixty-day-old greenhouse-grown plants were treated with 100 mg L^−1^ foliar chitosan alone or after pretreatment with LaCl₃, diphenyleneiodonium, cPTIO, or diethyldithiocarbamate, with inhibitor-only controls. Cytosolic Ca^2+^ -dependent fluorescence, NADPH oxidase activity, hydrogen peroxide, superoxide, NO, jasmone acid (JA), and jasmonoyl-isoleucine (JA-Ile) were monitored from 0 minutes to 7 d. Chitosan induced a temporally ordered response: Ca^2+^ fluorescence increased 3.01-fold within 15 minutes and returned to baseline by 3 hours; NADPH oxidase activity and hydrogen peroxide peaked at 3 hours; NO peaked at 6 hours; and JA and JA-Ile peaked at 12 hours. LaCl₃ suppressed all downstream responses, diphenyleneiodonium largely eliminated the oxidative burst, cPTIO abolished NO accumulation without affecting ROS production, and diethyldithiocarbamate selectively suppressed jasmone accumulation. These interventions support the sequence Ca^2+^→ NADPH oxidase-dependent ROS → NO → jasmone. Total targeted withanolides increased by 84% at day 7, including a 113.5% increase in withaferin A, whereas pathway inhibitors reduced this gain by 36%–49%. Enhanced antioxidant activity and improved glutathione and ascorbate redox states occurred without appreciable changes in lipid peroxidation, electrolyte leakage, photosynthesis, or biomass, indicating regulated redox signaling rather than injury. Untargeted LC–MS/MS revealed enrichment of steroid, terpenoid-backbone, and *α*-linolenic-acid metabolism and a 61.7% increase in the withanolide-precursor pool. Early signaling integrals predicted day-7 withanolide accumulation (*r* = 0.88–0.92), while structural equation modeling identified jasmone as the strongest direct predictor. Computational analyzes prioritized withaferin A and withanolide A as candidate IκB kinase *β* ligands, although biochemical validation is required. These findings show that signal timing organizes chitosan-induced withanolide biosynthesis in *W. somnifera*, with signaling dynamics predicting metabolic output. This pharmacological asymmetry provides strong directional evidence for pathway ordering, but it does not constitute definitive molecular proof of causality because inhibitor specificity is incomplete and no genetic or rescue experiment was performed.

## 1. Introduction

*Withania somnifera* (L.) Dunal, known as ashwagandha or Indian ginseng, is one of the most intensively used medicinal species of the Solanaceae family and occupies a central position in Ayurvedic, Unani, and folk medicine across South Asia, the Middle East, and parts of Africa.^1^ ^,^ ^2^ Root and leaf preparations have traditionally been prescribed as adaptogens, restoratives, and anti-inflammatory remedies, and the species is now one of the highest-value botanicals in the global nutraceutical trade, with formalized pharmacopoeial monographs and a rapidly expanding clinical literature on stress, sleep, and cognitive endpoints.^3-5^ The pharmacological reputation of the plant rests almost entirely on its withanolides, a family of C_28_ steroidal lactones built on an ergostane skeleton and elaborated by oxidation, lactonization, and glycosylation into more than 60 structurally distinct compounds.^6^ ^,^ ^7^ Four withanolides and two withanosides dominate both the commercial and pharmacological literature. Withaferin A, a highly oxygenated withanolide carrying a reactive *α*,*β*-unsaturated ketone in ring A, is the most biologically aggressive member of the family and has been reported to inhibit NF-κB signaling, disrupt intermediate filament assembly, inhibit the proteasome, and induce apoptosis in a wide range of tumor cell lines.^8-10^ Withanolide A is the compound most consistently associated with neuritogenic and neuroprotective activity, including dendrite regeneration and protection against amyloid-*β* toxicity.^11^ ^,^ ^12^ Withanone shows a striking selectivity in comparative assays, being cytotoxic to transformed cells at concentrations that normal fibroblasts tolerate, and is frequently studied together with withaferin A in leaf-derived extracts.^13^ Withanoside IV and withanoside V, the major glycowithanolides, are less cytotoxic but contribute substantially to the anxiolytic, antioxidant, and neuroprotective properties of root extracts, and their aglycone sominone is now regarded as an active metabolite in its own right.^14^ ^,^ ^15^

The chemical diversity of this family is not incidental. Because individual withanolides differ in the position of hydroxylation, in the presence of an epoxide or an enone, and in glycosylation state, they engage different molecular targets and show markedly different potency, selectivity, and bioavailability profiles.^16^ ^,^ ^17^ Therefore, a treatment that increases total withanolide content without altering the ratio between compounds is pharmacologically different from one that selectively enriches withaferin A or the withanosides. This makes compound-resolved quantification, rather than a single “total withanolide” number, a prerequisite for any meaningful evaluation of an elicitation strategy.^16^ Elicitors are best understood as external information signals rather than nutrients or growth regulators. A fungal cell-wall fragment, a bacterial flagellin peptide, or a chitosan oligomer carries no metabolic energy of consequence; what it carries is the pattern of a potential threat.^17^ ^,^ ^18^ Chitosan, the partially deacetylated derivative of chitin, is the most widely deployed elicitor in medicinal plant biotechnology precisely because it mimics a fungal signature while being inexpensive, non-toxic, biodegradable, and compatible with organic production systems.^19^ ^,^ ^20^ Perception occurs at the plasma membrane. Chitin and chitosan oligomers are bound by lysin-motif (LysM) receptor-like kinases and associated receptor-like proteins, which then recruit co-receptors and initiate transphosphorylation; in parallel, the polycationic character of chitosan allows it to interact directly with the anionic phospholipid face of the membrane, altering membrane potential and permeability.^21-23^ Both routes converge on the same early output: a rapid, transient change in ion fluxes across the plasma membrane.

What follows is the activation of a secondary-messenger network. Cytosolic Ca^2+^ elevation, activation of respiratory burst oxidase homolog (RBOH) NADPH oxidases, mitogen-activated protein kinase cascades, nitric oxide (NO) production, and phytohormone biosynthesis together constitute a signaling layer that decodes the elicitor stimulus and translates it into transcriptional and enzymatic reprogramming.^24-26^ Downstream, carbon and reducing power are redirected from primary growth metabolism toward the phenylpropanoid, terpenoid, alkaloid, or steroidal-lactone pathways, depending on the species.^27^ ^,^ ^28^ It is essential to distinguish this defensive signaling from irreversible stress injury. The same molecules–Ca^2+, H2O2, and^ NO–that carry information at low, transient concentrations become agents of oxidative and nitrosative damage when they are produced in excess or fail to return to baseline.^29^ ^,^ ^30^ At elicitor doses that exceed the signaling window, lipid peroxidation, membrane leakage, chlorophyll loss, and growth arrest appear, and any increase in specialized metabolites is then a symptom of damage rather than a controlled metabolic decision.^31^ A credible elicitation study must therefore report injury markers alongside metabolite gains.

Cytosolic free Ca^2+^ is the fastest and most general second messenger in plant elicitor responses. Within seconds to a few minutes of elicitor perception, Ca^2+^ enters the cytosol through plasma membrane channels, including cyclic nucleotide-gated channels and glutamate receptor-like channels, and is released from internal stores, producing a transient that typically resolves within tens of minutes as Ca^2+^-ATPases and exchangers restore the resting concentration.^32-34^ The information content of these transients lies in its shape. The amplitude, duration, number of oscillations, and subcellular location of the rise together constitute a “calcium signature” that differs between stimuli and is decoded by calmodulins, calcineurin B-like proteins, CBL-interacting protein kinases, and calcium-dependent protein kinases.^35-37^ Therefore, two elicitors that produce the same peak amplitude but different durations can produce entirely different transcriptional outputs. A major downstream consequence of the Ca^2+^ transient is the activation of redox processes. RBOH proteins carry EF-hand motifs in their *N*-terminal cytosolic region and are additionally phosphorylated by calcium-dependent protein kinases, so their activity is directly gated by cytosolic Ca^2+^.^38^ ^,^ ^39^ Blocking Ca^2+^ influx with lanthanide salts such as LaCl_3_ therefore predictably attenuates the subsequent oxidative burst in most systems examined.^35^ The practical limitation of the existing literature on *W. somnifera* is that the Ca^2+^ step is usually inferred rather than observed. Studies that sample at 24 hours or 48 hours—after the transient has long resolved—cannot detect it at all, and studies that report only a single “elicited versus control” contrast cannot distinguish a brief physiological transient from a sustained pathological rise.^36^

The oxidative burst that follows elicitor perception is generated principally by plasma-membrane NADPH oxidases, which transfer electrons from cytosolic NADPH to extracellular oxygen to form superoxide; superoxide is then dismutated, spontaneously or by superoxide dismutase, to hydrogen peroxide.^37^ ^,^ ^40^ H_2_O_2_ is comparatively stable, uncharged, and able to cross membranes through aquaporins, which makes it well suited to act as a diffusible messenger between compartments and between cells.^41^ H_2_O_2_ functions through the reversible oxidation of cysteine thiols in transcription factors, phosphatases, and redox-sensitive enzymes, thereby modulating gene expression rather than destroying cellular structures.^42^ ^,^ ^43^ The transition from signal to damage is quantitative and kinetic: what matters is not whether H_2_O_2_ rises, but how far it rises and how quickly the antioxidant system returns it to baseline.

Nitric oxide is produced in parallel following elicitor perception through nitrate reductase-dependent and oxidative routes whose relative contribution remains debated in plants.^44^ ^,^ ^45^ NO acts largely through *S*-nitrosylation of cysteine residues, tyrosine nitration, and metal nitrosylation, and its targets overlap extensively with those of H_2_O_2_.^46^ ROS and NO cooperate and antagonize each other in a concentration-dependent manner. NO can *S*-nitrosylate RBOHD at Cys890, inhibiting superoxide production and thereby limiting the burst, while ROS can promote NO synthesis through the activation of nitrate reductase; the two also react to form peroxynitrite.^47-49^ The net outcome—amplification, damping, or damage—depends on the relative timing of the two signals, which is precisely the information that single-time-point sampling destroys. At excessive concentrations, the same chemistry generates lipid peroxidation, protein carbonylation, and irreversible nitrosative injury.^50^

Jasmonic acid and its bioactive conjugate, jasmonoyl-isoleucine, are canonical hormonal integrators of defense responses. JA-Ile promotes the interaction of the receptor COI1 with JAZ repressor proteins, targeting them for degradation and releasing MYC-type and other transcription factors that drive defense and specialized-metabolism gene expression.^51-53^ Jasmonate biosynthesis is downstream of and responsive to redox signals. Lipoxygenase activity, the release of *α*-linolenic acid from galactolipids, and the activity of allene oxide synthase are all sensitive to the cellular redox state, and both H_2_O_2_ and NO have been reported to promote jasmonate accumulation in several species.^54-56^ This positions jasmonate as a plausible relay that converts a short-lived redox event into a sustained transcriptional program. In *W. somnifera*, methyl jasmonate is the single most effective known elicitor of withanolide accumulation in both whole plants and hairy-root and cell-suspension cultures, and jasmonate treatment upregulates squalene epoxidase, cycloartenol synthase, sterol methyltransferase, and several cytochrome P450 genes of the withanolide pathway.^57-59^ The position of endogenous jasmonate within a chitosan-triggered cascade remains unresolved. Reports differ as to whether jasmonate acts upstream of the oxidative burst, downstream of it, or in a parallel branch, and the question has not been settled in this species with inhibitor-based evidence.^60^ ^,^ ^61^

Despite the large literature on the elicitation of withanolides, five limitations remain. First, elicitor responses are usually measured at only one or two time points, most often 24 hours or 48 hours, which is far too late to capture Ca^2+^ and too coarse to order ROS, NO, and jasmonate. Second, antioxidant and withanolide changes are reported side by side without any evidence that one causes the other; therefore, the signal order is assumed rather than demonstrated. Third, inhibitor- and scavenger-based causal tests, which are routine in model species signaling studies, are rarely applied to medicinal plant elicitation studies. Fourth, signal kinetics and untargeted metabolomics are rarely integrated into the same experiment; therefore, it is unknown whether early signaling features predict the later metabolic phenotype. Fifth, computational pharmacology is often performed on compounds selected from the literature rather than on those that the treatment actually increased, which severs the link between the agronomic intervention and the pharmacological claim.^62-65^ This study was designed to close these gaps in a single, fully time-resolved experiment. The specific objectives of this study were as follows: (i) to characterize the temporal dynamics of cytosolic Ca^2+, ROS, NO,^ and jasmonate following chitosan treatment of intact *W. somnifera* plants; (ii) to determine the functional hierarchy among these components by pharmacological disruption with LaCl_3_, DPI, cPTIO, and DIECA; (iii) to identify elicitor-responsive metabolic pathways and withanolides by targeted and untargeted LC-MS/MS; (iv) to associate early signal features with subsequent withanolide accumulation using area-under-the-curve, time-lagged correlation, and structural equation modeling; and (v) to assess the interaction of the major elicitor-responsive withanolides with one biologically justified therapeutic target class.

## 2. Materials and methods

### 2.1. Experimental site, plant material, and growth conditions

The experiment was conducted in the research glasshouse of the Department of Zoology, The Women University Multan, Mattital Campus, Multan, Pakistan (30.1959° N, 71.4681° E; 122 m above sea level) between February 3 and May 28, 2025, and was repeated in full as a second independent run beginning on March 11, 2025. Certified seeds of *Withania somnifera* (L.) Dunal (accession WS-NARC-11) were obtained from the National Institute for Genomics and Advanced Biotechnology, National Agricultural Research Center, Islamabad. The botanical identity was confirmed by a plant taxonomist using floral and fruit characteristics, and a voucher specimen (WS-WUM-2025-118) was deposited in the Department of Zoology, The Women University Multan. Seeds were surface-sterilized in 0.1% (w/v) HgCl_2_ for 3 minutes, rinsed five times with sterile distilled water, and sown in germination trays containing autoclaved sand. Trays were kept at 25 ± 1 °C in darkness until radicle emergence (6–8 d) and then transferred to the glasshouse bench. Fifteen days after sowing, uniform seedlings at the two-true-leaf stage were transplanted singly into 6 L plastic pots (22 cm top diameter) containing 5.5 kg of a sandy-loam soil: sphagnum peat: washed river sand mixture (2:1:1, v/v/v). The substrate had pH 7.4 (1:2.5 soil: water), electrical conductivity 1.21 dS m^−1^, organic matter 1.38%, 9.6 mg kg^−1^ available *P*, and 176 mg kg^−1^ extractable K.

Glasshouse conditions were maintained at 26 ± 2 °C during the day and 18 ± 2 °C at night, 62 ± 5% relative humidity, and a 14 hours photoperiod under natural light supplemented by cool-white LED panels to a mean canopy photosynthetic photon flux density of 620 ± 40 μmol m^−2^ s^−1^. Air temperature, relative humidity, and PPFD were logged at 15 minutes intervals beside the canopy throughout the sampling period and are reported with the dataset (mean 26.0 ± 0.6 °C, 61.9 ± 2.5% RH, 620 ± 37 μmol m^−2^ s^−1^). Pots were irrigated to 70%–75% of field capacity by daily gravimetric weighing, and soil moisture at sampling averaged 72.0% ± 1.8% of field capacity. A half-strength Hoagland solution (250 mL per pot) was applied fortnightly from day 20 to day 50. Pots were re-randomized weekly to remove positional effects. All treatments were applied to plants at 60 d after sowing, at the pre-flowering vegetative stage, when plants carried 9–11 fully expanded leaves.

### 2.2. Elicitor preparation, dose optimization and treatment design

Low-molecular-weight chitosan (50–190 kDa; degree of deacetylation ≥85%; Sigma-Aldrich 448869) was used throughout. A 1 g L^−1^ stock was prepared by dispersing chitosan in 1% (v/v) acetic acid with continuous magnetic stirring at 40 °C for 4 hours until fully dissolved, cooled to room temperature, adjusted to pH 5.6 ± 0.1 with 1 M NaOH, made up to volume with deionized water, and filtered through Whatman No. 1 paper. Working solutions were prepared fresh on the day of use and amended with 0.02% (v/v) Tween-20 as a surfactant. The vehicle control was prepared identically but without chitosan (1% acetic acid adjusted to pH 5.6 with 0.02% Tween-20), so that pH, ionic strength, and surfactant were matched across treatments. A preliminary dose-response experiment (six plants per dose, repeated twice) was run before the main experiment. Plants received 0, 25, 50, 100, 200, or 400 mg L^−1^ chitosan and were assessed 7 d later for total targeted withanolides, electrolyte leakage, malondialdehyde, and shoot dry weight. The lowest dose that produced a near-maximal withanolide response without a measurable increase in membrane injury or a loss of biomass was selected for the main experiment.

Solutions were applied as a combined foliar spray and soil drench: 15 mL was sprayed onto the shoot with a hand atomizer until run-off, and 10 mL was pipetted onto the substrate surface within the root zone, giving 25 mL per plant. All applications were made between 08:00 and 08:45 to minimize diurnal variation, and time zero was recorded as the moment the spray was completed on each individual plant. Ten treatments were used (Table 1). T01 was the vehicle control, and T02 was chitosan at 100 mg L^−1^. Four inhibitor combinations, T03-T06, consisted of chitosan preceded by a 60 minutes pre-treatment with 5 mM LaCl_3_ (plasma-membrane Ca^2+^-channel blocker), 10 μM diphenyleneiodonium (DPI; flavoenzyme/NADPH oxidase inhibitor), 200 μM cPTIO (NO scavenger) or 1 mM sodium diethyldithiocarbamate (DIECA; lipoxygenase inhibitor that blocks jasmonate biosynthesis). Four inhibitor-only controls, T07-T10, received the same pre-treatment followed by the vehicle instead of chitosan, and are the essential test of whether any observed effect belongs to the inhibitor itself. Inhibitors were applied by the same spray-plus-drench route in the same total volume; DPI was first dissolved in dimethyl sulfoxide and diluted so that the final DMSO concentration never exceeded 0.05% (v/v), which was matched in all other treatments including the controls.

The causal interpretation of the inhibitor experiment was based on directional asymmetry rather than inhibition alone. An upstream step was considered functionally required for a downstream response when blocking that step significantly attenuated the downstream signal, whereas inhibition of a downstream step left the upstream signal largely unchanged. Inhibitor-only controls were used to exclude direct treatment effects. Because LaCl_3_, DPI, cPTIO, and DIECA are pharmacological probes with recognized off-target potential, these criteria were used to infer pathway dependency and order, not to claim irreversible molecular causation; definitive causality would require orthogonal rescue experiments and/or genetic perturbation of the corresponding signaling components.

**Table 1.** Treatment structure of the main experiment. All treatments were applied to 60-d-old plants as a combined foliar spray (15 mL) and soil drench (10 mL) solution. Inhibitors were applied 60 minutes before adding the elicitor or vehicle.

Code	Treatment	Chitosan (mg L^−1^)	Inhibitor	Concentration	Target step/purpose
T01	Vehicle control	0	None	−	Baseline; matched pH, acetate, and surfactant
T02	Chitosan	100	None	−	Elicitor reference treatment
T03	LaCl_3_ + chitosan	100	LaCl_3_	5 mM	Plasma-membrane Ca^2+^-channel blockade
T04	DPI + chitosan	100	DPI	10 μM	NADPH oxidase (flavoenzyme) inhibition
T05	cPTIO + chitosan	100	cPTIO	200 μM	Nitric oxide scavenging
T06	DIECA + chitosan	100	DIECA	1 mM	Lipoxygenase/jasmonate biosynthesis inhibition
T07	LaCl_3_ only	0	LaCl_3_	5 mM	Inhibitor-only control
T08	DPI only	0	DPI	10 μM	Inhibitor-only control
T09	cPTIO only	0	cPTIO	200 μM	Inhibitor-only control
T10	DIECA only	0	DIECA	1 mM	Inhibitor-only control

CHT, chitosan; cPTIO, 2-(4-carboxyphenyl)-4,4,5,5-tetramethylimidazoline-1-oxyl-3-oxide; DIECA, sodium diethyldithiocarbamate; DPI, diphenyleneiodonium. Twelve sampling times × four biological replicates × two independent runs were used for each treatment (960 plants in total).

Each treatment × time-point combination was represented by four independent biological replicates, each replicate being one separately potted plant, and the entire experiment was repeated as two independent runs, resulting in eight biological replicates per mean. Because sampling was destructive, a separate set of plants was grown for every time point: 10 treatments × 12 time points × 4 replicates × 2 runs = 960 plants were grown. The plants were arranged in a completely randomized design for each run.

### 2.3. Sampling scheme

Tissue was collected at 12 time points: 0 minute (immediately before treatment), 5, 15, and 30 minutes, and 1, 3, 6, 12, 24, 48, and 72 hours and 7 d after elicitation. The youngest fully expanded leaf pair of each plant was harvested, and the leaf material was immediately split into three portions: one portion was used fresh for fluorescence and physiological assays, one was snap-frozen in liquid nitrogen and stored at −80 °C for enzyme, hormone, and metabolite analysis, and one was oven-dried at 45 °C to constant weight for withanolide quantification and dry-weight determination. The interval between excision and freezing never exceeded 45 seconds.

### 2.4. Cytosolic Ca^2+^-dependent fluorescence

Relative changes in cytosolic Ca^2+^ were monitored using the acetoxymethyl ester probe Fluo-4 AM. Leaf discs (6 mm diameter, four per plant) were cut with a cork borer, vacuum-infiltrated for 2 minutes in loading buffer (10 mM MES-KOH pH 5.8, 5 mM KCl, 0.1 mM CaCl_2_) containing 5 μM Fluo-4 AM and 0.02% Pluronic F-127, and incubated in the dark at 25 °C for 60 minutes. The discs were then washed three times for 20 minutes in probe-free buffer to allow complete de-esterification, and fluorescence was read in a black 96-well microplate on a fluorescence plate reader at 494 nm excitation and 516 nm emission with a 495 nm cutoff filter. Unloaded discs from the same leaf were read on every plate, and their signals were subtracted as tissue autofluorescence; wells containing buffer only were used as instrument blanks. Because ester-loaded probes cannot be reliably calibrated in intact tissue, all values are reported as background-corrected relative fluorescence units and, in the figures, as the fold change relative to the reading taken on the same plant material immediately before treatment (time 0). No claims were made regarding the absolute cytosolic Ca^2+^ concentration.

### 2.5. Reactive oxygen species, NADPH oxidase activity and membrane-injury markers

Hydrogen peroxide was quantified using the potassium iodide method. Frozen leaf tissue (0.2 g) was homogenized in 2 mL of ice-cold 0.1% (w/v) trichloroacetic acid, centrifuged at 12 000 × g for 15 minutes at 4 °C, and 0.5 mL of supernatant was mixed with 0.5 mL of 10 mM potassium phosphate buffer (pH 7.0) and 1 mL of 1 M KI. After 60 minutes in the dark, absorbance was measured at 390 nm, and the concentration was obtained from a standard curve prepared with authentic H_2_O_2_. Superoxide generation was measured as the hydroxylamine-dependent formation of nitrite: tissue extract was incubated with 1 mM hydroxylamine hydrochloride, and the nitrite formed was determined colorimetrically at 530 nm after reaction with sulfanilamide and *N*-(1-naphthyl)ethylenediamine, using a NaNO_2_ standard curve.

NADPH oxidase activity was assayed in a plasma membrane-enriched microsomal fraction obtained by differential centrifugation (10 000 × g for 15 minutes, then 100 000 × g for 45 minutes at 4 °C). The pellet was resuspended in 50 mM Tris-HCl (pH 7.5) containing 250 mM sucrose and 1 mM dithiothreitol, and activity was measured as the superoxide-dependent reduction of nitroblue tetrazolium at 530 nm after the addition of 100 μM NADPH; the DPI-inhibitable fraction of the rate is reported, and activity is expressed as nmol O_2_^•-^ g^−1^ FW min^−1^. Protein was determined using the Bradford method with bovine serum albumin as the standard. For localization, additional leaves were stained with 1 mg mL^−1^ 3,3′-diaminobenzidine (pH 3.8) for H_2_O_2_ and 0.1% (w/v) nitroblue tetrazolium in 10 mM phosphate buffer for superoxide, vacuum-infiltrated for 5 minutes, incubated in darkness for 8 hours, cleared in boiling 95% ethanol, and photographed under a stereomicroscope. Two injury markers were measured at every time point so that signaling could be separated from damage. Lipid peroxidation was estimated as malondialdehyde by the thiobarbituric acid reaction, with absorbance read at 532 nm and corrected for non-specific turbidity at 600 nm using an extinction coefficient of 155 mM^−1^ cm^−1^. Electrolyte leakage was measured by floating 10 leaf discs in 20 mL deionized water for 4 hours at 25 °C (EC_1_), autoclaving at 121 °C for 20 minutes, cooling and re-reading (EC_2_), and expressing leakage as 100 × EC_1_/EC_2_.

### 2.6. Nitric oxide determination

NO was detected using DAF-FM diacetate. Leaf discs were loaded in 10 mM Tris-HCl (pH 7.4) containing 10 μM DAF-FM DA for 30 minutes in darkness at 25 °C, washed three times in probe-free buffer, and read at 495 nm excitation and 515 nm emission. Autofluorescence of unloaded discs was subtracted, and values are expressed as relative fluorescence units and as the fold change relative to time 0. Three controls were used to establish specificity. Discs treated with 200 μM cPTIO before probe loading served as the NO-scavenged negative control and quenched more than 85% of the elicitor-dependent signal; discs treated with the NO donor sodium nitroprusside (100 μM) served as the positive control; and probe-free discs were controlled for tissue autofluorescence. As an independent chemical confirmation, tissue nitrite was quantified by the Griess reaction at 540 nm in a subset of samples at 0, 3, 6, and 12 hours, and gave the same treatment ranking as the fluorescence data.

### 2.7. Jasmonate quantification

Jasmonic acid and jasmonoyl-isoleucine were quantified using LC-MS/MS. Frozen tissue (100 mg) was ground under liquid nitrogen and extracted overnight at 4 °C in 1 mL of methanol: water: formic acid (75:20:5, v/v/v) containing 10 ng of d_5_-JA and 10 ng of d_5_-JA-Ile as internal standards. Extracts were centrifuged at 14 000 × g for 10 minutes, re-extracted once, combined, evaporated under nitrogen, and reconstituted in 200 μL of 30% methanol. Purification was performed using a reversed-phase solid-phase extraction cartridge conditioned with methanol and water, washed with 5% methanol, and eluted with 80% methanol. Separation was performed on a C18 column (2.1 × 100 mm, 1.8 μm) at 40 °C with 0.1% formic acid in water (A) and acetonitrile (B) at 0.3 mL min^−1^, using a gradient from 20% to 95% B over 9 minutes. Detection was performed using electrospray ionization in negative mode with multiple-reaction monitoring (JA, *m/z* 209 → 59; JA-Ile, *m/z* 322 → 130; deuterated analogs monitored in parallel). Quantification was performed against eight-point calibration curves, and concentrations are expressed as ng g^−1^ FW. Methyl jasmonate was monitored in the positive mode in a subset of samples but remained below the limit of quantification and is not reported.

### 2.8. Antioxidant enzymes and redox ratios

Frozen tissue (0.3 g) was homogenized in 3 mL of ice-cold 50 mM potassium phosphate buffer (pH 7.8) containing 1 mM EDTA, 1 mM dithiothreitol, and 2% (w/v) polyvinylpolypyrrolidone, and the supernatant obtained after centrifugation at 15 000 × g for 20 minutes at 4 °C was used for all enzyme assays. For ascorbate peroxidase, the buffer additionally contained 2 mM ascorbate. Superoxide dismutase was assayed as the inhibition of the photochemical reduction of nitroblue tetrazolium at 560 nm, with one unit defined as the enzyme quantity causing 50% inhibition. Catalase was measured as the decline in absorbance at 240 nm during the consumption of 15 mM H_2_O_2_ (*ε* = 39.4 mM^−1^ cm^−1^). Ascorbate peroxidase was measured as ascorbate oxidation at 290 nm (*ε* = 2.8 mM^−1^ cm^−1^) in the presence of 0.5 mM ascorbate and 0.1 mM H_2_O_2_. Glutathione reductase was measured as NADPH oxidation at 340 nm (*ε* = 6.22 mM^−1^ cm^−1^) in the presence of 0.5 mM oxidized glutathione. Activities are expressed on a protein basis. Reduced and oxidized glutathione were determined by the 5,5′-dithiobis-(2-nitrobenzoic acid)-glutathione reductase recycling assay at 412 nm, with GSSG measured after derivatization of GSH with 2-vinylpyridine and GSH obtained by difference. Ascorbate and dehydroascorbate were determined at 525 nm after reduction with dithiothreitol, with dehydroascorbate obtained as the difference between total and reduced ascorbate. The GSH/GSSG and AsA/DHA ratios are reported because they, rather than absolute pool sizes, indicate the direction in which the cellular redox poise has moved.

### 2.9. Physiological and growth measurements

Relative water content was calculated as 100 × (FW − DW)/(TW − DW), where turgid weight was obtained after floating leaf discs on deionized water for 4 hours in darkness at 4 °C and dry weight after 48 hours at 70 °C. Chlorophyll status was recorded non-destructively as SPAD units on the third fully expanded leaf, averaging six readings per plant. Net photosynthetic rate and stomatal conductance were measured on the youngest fully expanded leaf with a portable infrared gas analyzer between 09:30 and 11:30, at 1200 μmol m^−2^ s^−1^ photosynthetic photon flux density, 400 μmol mol^−1^ reference CO_2_, leaf temperature 26 °C, and 60% relative humidity in the cuvette. Maximum quantum efficiency of photosystem II (*F*_v_/*F*_m_) was measured with a portable fluorometer after 30 minutes of dark adaptation using leaf clips. Shoot dry weight was recorded after drying the entire above-ground biomass at 70 °C for 72 hours.

### 2.10. Targeted withanolide quantification and method validation

Dried leaf material was milled to pass through a 0.5 mm sieve. Powder (100 mg) was extracted three times with 3 mL of methanol by ultrasonication for 30 minutes at 35 °C, and the pooled extract was centrifuged at 10 000 × g for 10 minutes, evaporated to dryness, reconstituted in 1 mL of methanol, and filtered through a 0.22 μm PTFE membrane before injection. Withaferin A, withanolide A, withanone, withanoside IV, and withanoside V were quantified against authentic reference standards (purity ≥ 95%, verified by ^1^H NMR) on a triple-quadrupole LC-MS/MS system with a C18 column (2.1 × 100 mm, 1.8 μm) held at 35 °C. The mobile phase was 0.1% formic acid in water (A) and acetonitrile (B) at 0.3 mL min^−1^ with a 20%–95% B gradient over 14 minutes. Detection used electrospray ionization in positive mode with multiple-reaction monitoring; two transitions were monitored per analyte, the more intense being used for quantification and the second for confirmation. Method performance was validated before sample analysis (Table 2). Calibration curves were linear over 0.05–50 μg mL^−1^ with coefficients of determination ≥ 0.9985. The limits of detection and quantification, calculated as 3.3σ/S and 10σ/S from the calibration residuals, ranged from 3.1 to 9.4 ng mL^−1^ and 9.5 to 28.5 ng mL-1, respectively. Recovery, determined by spiking blank matrix at three concentrations, was 92.6%–101.4%. Intra-day precision (six injections within one day) and inter-day precision (three consecutive days) were ≤3.2% and ≤5.1%, respectively. Results are expressed as mg g^−1^ dry weight, and the total targeted withanolide content is the arithmetic sum of the five analytes.

**Table 2.** Validation of the targeted LC-MS/MS method used for withanolide quantification.

Analyte	MRM transition (*m/z*)	Range (μg mL^−1^)	*R* ^2^	LOD (ng mL^−1^)	LOQ (ng mL^−1^)	Recovery (%)	Intra-day RSD (%)	Inter-day RSD (%)
Withaferin A	471.3 → 281.2	0.05–50	0.9995	3.1	9.5	99.2 ± 2.1	1.8	3.2
Withanolide A	471.3 → 109.1	0.05–50	0.9992	3.6	11.0	98.4 ± 2.6	2.1	3.7
Withanone	471.3 → 263.2	0.05–50	0.9989	4.2	12.7	101.4 ± 3.0	2.4	4.1
Withanoside IV	783.4 → 471.3	0.05–50	0.9987	8.1	24.6	94.8 ± 3.4	2.9	4.8
Withanoside V	767.4 → 455.3	0.05–50	0.9985	9.4	28.5	92.6 ± 3.6	3.2	5.1

LOD, limit of detection (3.3σ/S); LOQ, limit of quantification (10σ/S); MRM, multiple reaction monitoring; RSD, relative standard deviation. Recovery was determined by spiking a blank matrix at three concentrations (*n* = 6 at each level).

### 2.11. Untargeted metabolomics and metabolomic data analysis

Untargeted profiling used the same methanolic extracts diluted 1:5. Chromatography was performed on a C18 column (2.1 × 100 mm, 1.8 μm) with a 22 minutes gradient of 5%–95% acetonitrile in 0.1% formic acid at 0.3 mL min^−1^, and detection was performed using a quadrupole time-of-flight mass spectrometer operated in both positive and negative electrospray modes over *m/z* 100–1200, with data-dependent MS/MS acquisition on the four most intense precursors per cycle at collision energies of 10, 20, and 40 eV. Quality control followed accepted metabolomics practices. A pooled quality control sample was prepared by combining equal aliquots of every study sample and was injected after every 10 study samples. Extraction and solvent blanks were injected at the start, middle, and end of each batch. A mixture of retention-time standards was injected at the start of each batch. The injection order of study samples was randomized within and across batches to prevent confounding of treatment with run order. Samples from both experimental runs were analyzed in two batches (LCMS_R1 and LCMS_R2), each preceded by five conditioning injections. Raw files were converted to mzML and processed using an open-source workflow for peak picking, chromatogram deconvolution, isotope grouping, alignment, and gap filling. Features present in blanks at more than 20% of the mean study sample intensity were discarded, as were features with a relative standard deviation above 30% in the pooled quality control injections or detected in fewer than 70% of samples in at least one treatment group. The intensities were normalized to the total useful signal, log_10_-transformed, and Pareto-scaled. After filtering, 3913 ± 219 features were retained for each sample.

Annotation confidence was reported according to the Metabolomics Standards Initiative. Level 1 annotations were assigned only where the retention time and MS/MS spectra matched an authentic standard analyzed in the same sequence (the five targeted withanolides, JA, and JA-Ile). Level 2 annotations were assigned based on accurate mass (≤5 ppm), isotope pattern, and MS/MS match to public spectral libraries. Level 3 annotations indicate only compound class assignments. The databases and libraries used were METLIN, MassBank, GNPS, the KNApSAcK plant database, and an in-house withanolide spectral library. Multivariate analysis used principal component analysis on Pareto-scaled data for unsupervised structure, hierarchical clustering with Euclidean distance and Ward linkage for sample and feature grouping, and heat maps of row-standardized values for visualization. Partial least-squares discriminant analysis was used only in a confirmatory role, with the model validated by seven-fold cross-validation and 1000 random permutations. Differential accumulation was tested using linear models with Benjamini–Hochberg false discovery rate correction, with features considered to be differentially accumulated at *q*-value < 0.05 and log_2_ fold change > 1. Pathway enrichment and impact were computed using the *Solanaceae* Reference Pathway Library. Correlation networks linking signaling variables to metabolite features were constructed using Spearman coefficients thresholded at *p* > 0.6 and *q* < 0.05. For pathway-level interpretation, Level 1 identities were treated as chemically confirmed, Level 2 assignments as putative compound annotations, and Level 3 assignments as tentative compound class evidence. Pathway enrichment based on Level 2/3 features was used to support pathway-level patterns rather than to assert the definitive identity of each metabolite. Where isomeric compounds could not be distinguished by accurate mass and library MS/MS alone, the annotation was retained at Level 2 or 3 and was not promoted to Level 1 without an authentic standard and a retention time match. Untargeted LC–MS/MS analysis quantifies relative metabolite abundance at defined sampling times but does not directly measure metabolic flux. Increases in sterol precursors, pathway enrichment, and withanolide end products were interpreted as evidence of coordinated pathway activation and increased precursor availability rather than direct proof of a higher reaction rate through the pathway. Direct flux estimation requires isotope-tracer experiments (for example, 13C-labeled precursor incorporation), metabolic flux analysis, or comparable kinetic measurements, which are beyond the scope of the present study.

### 2.12. Computational target assessment

Only withanolides that were both experimentally detected and significantly increased by chitosan were carried forward so that the computational work described the compounds that were actually produced. Ligand structures were retrieved from PubChem, protonated at pH 7.4, energy-minimized using the MMFF94 force field, and saved with Gasteiger charges. One biologically justified target class was selected: withaferin A is most consistently reported to act through inhibition of the NF-κB activation module, and the human IκB kinase *β* (IKKβ) catalytic domain was used (PDB 4KIK, 2.30 Å). The structure was validated before use: Ramachandran analysis placed 96.4% of residues in the favored regions, missing side chains were repaired, water molecules and heteroatoms other than the co-crystallized ligand were removed, hydrogens were added, and Kollman charges were assigned. The docking protocol was validated by redocking the co-crystallized reference ligand into the prepared receptor. The pose was reproduced with a heavy-atom root-mean-square deviation of 0.94 Å from the crystallographic pose, below the 2.0 Å acceptance threshold. Docking then used a grid box of 24 × 24 × 24 Å centered on the ATP-binding cleft, exhaustiveness 32, and ten poses per ligand, with the highest-scoring pose retained. Interaction residues were identified and classified as hydrogen bonds, hydrophobic contacts, or *π*-alkyl or van der Waals contacts.

The two top-ranked complexes and the apo protein were subjected to 100 ns all-atom molecular-dynamics simulation with the CHARMM36m force field and CGenFF ligand parameters in a TIP3P water box with 10 Å padding, neutralized with 0.15 M NaCl. After energy minimization, 100 ps NVT and 1 ns NPT equilibration at 310 K and 1 bar, production runs used a 2 fs time step with LINCS constraints and particle-mesh Ewald electrostatics. Backbone root-mean-square deviation, per-residue root-mean-square fluctuation, radius of gyration, solvent-accessible surface area, and hydrogen-bond occupancy were computed from the trajectories. Binding free energies were estimated using the molecular mechanics generalized-Born surface-area method on 200 frames drawn from the last 20  ns. In silico ADMET properties, such as gastrointestinal absorption, blood-brain barrier permeation, *P*-glycoprotein substrate status, cytochrome P450 inhibition, aqueous solubility, total polar surface area, Lipinski compliance, AMES mutagenicity, and predicted hepatotoxicity, were obtained from established web-based predictors. All computational results are reported as interaction and property *predictions*; no claim of therapeutic efficacy is made based on docking or simulation alone.

### 2.13. Statistical analysis

Analyzes were performed using R 4.3.2. Time-course variables were analyzed using linear mixed-effects models containing treatment, time, and their interaction as fixed effects and experimental run as a random effect, fitted by restricted maximum likelihood; degrees of freedom were approximated using the Kenward–Roger method. Because the design used destructive sampling, plants were independent at each time point, and this is reflected in the model structure. Factorial two-way analysis of variance was additionally used to report treatment, time, and treatment × time effects in a form directly comparable to the earlier literature. Normality and homoscedasticity were checked on model residuals with the Shapiro‒Wilk and Levene tests; jasmonate and fluorescence variables were log-transformed before analysis, and the results were back-transformed for presentation. Multiple comparisons used Tukey's honestly significant difference test at *p* < 0.05, and metabolomic features were corrected by the Benjamini‒Hochberg procedure at *q* < 0.05. Signal magnitude was summarized for each individual plant lineage as the area under the concentration-time curve computed by the trapezoidal rule over the biologically relevant window for each signal: 0–1 hours for Ca^2+^, 0–12 hours for H_2_O_2_ and NADPH oxidase, 0–24 hours for NO, and 0–72 hours for JA and JA-Ile. Peak amplitude, time to peak, and time to return within 10% of baseline were extracted from the same curves. Relationships between early signals and later metabolites were examined by Pearson correlation of area-under-the-curve values against day-7 metabolite concentrations and by time-lagged correlation in which the signal value at each individual sampling time was correlated with the day-7 withanolide content. A structural equation model was fitted with the *lavaan* package to test the hypothesized sequence chitosan → Ca^2+^ → NADPH oxidase-dependent ROS → NO → jasmonate → withanolide accumulation, with direct paths from Ca^2+^ and ROS to withanolides retained to allow jasmonate-independent routes. Variables were *z*-standardized, the model was estimated by maximum likelihood, and the fit was judged by the comparative fit index, Tucker–Lewis index, root-mean-square error of approximation, and standardized root-mean-square residual. All values in the text and tables are means ± standard deviation of eight biological replicates unless stated otherwise.

## 3. Results

### 3.1. Elicitor-dose optimization

**Figure 1.**
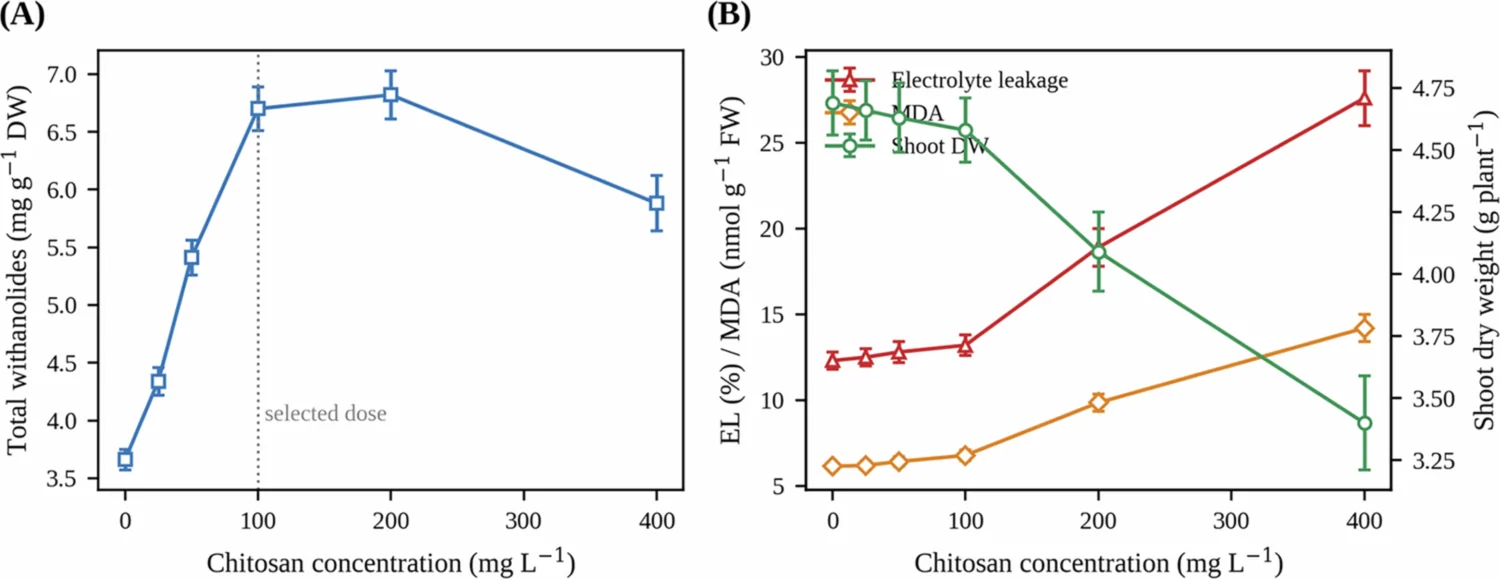
Chitosan dose optimization in *W. somnifera* seven days after a single foliar-plus-drench application. (A) Total targeted withanolide content. (B) Electrolyte leakage and malondialdehyde (left axis) and shoot dry weight (right axis). The dotted line marks the 100 mg L^−1^ dose selected for the main experiment. Values are means ± SD of six plants per dose from two independent runs.

Chitosan increased the total targeted withanolide content in a dose-dependent but saturating manner (Figure 1A; Table 3). Relative to the vehicle control (3.66 ± 0.09 mg g^−1^ DW), doses of 25, 50, and 100 mg L^−1^ raised total withanolides by 18.6%, 47.8%, and 83.1%, respectively, whereas 200 mg L^−1^ gave no further gain (+86.3%, statistically indistinguishable from 100 mg L^−1^, *p* = 0.41) and 400 mg L^−1^ produced a lower content than 100 mg L^−1^ (+60.7%). Injury markers separated the two halves of the dose series cleanly (Figure 1B). Up to 100 mg L^−1^, electrolyte leakage (12.3 → 13.2%), malondialdehyde (6.14 → 6.78 nmol g^-1^ FW), and shoot dry weight (4.69 → 4.58 g plant^−1^) were statistically unchanged. At 200 mg L^−1^, electrolyte leakage rose by 54% and malondialdehyde by 60%, and shoot dry weight fell by 12.8%; at 400 mg L^−1^ the corresponding values were +124%, +131%, and −27.5%. The decline in withanolide content at the highest dose therefore coincided with clear tissue damage rather than with enhanced metabolism. A dose of 100  mg L^−1^ was accordingly selected as the biologically effective, non-toxic dose and was used in all subsequent experiments. This concentration produced 96.3% of the maximum observed withanolide response while leaving every injury and growth indicator within the control range. The SEM was specified a priori to evaluate whether the observed covariance structure was consistent with the biologically hypothesized signaling sequence; it was not treated as an independent experimental test of causation. Standardized path coefficients therefore represent conditional statistical associations within the specified model, after accounting for the other included paths, and should not be interpreted as proof that changing one node necessarily causes the estimated change in the next. Causal interpretation was reserved for the convergence of SEM with temporal precedence and the directional pharmacological perturbation experiments, while acknowledging that unmeasured confounding, feedback, alternative model structures, and inhibitor off-target effects cannot be excluded.

**Table 3.** A preliminary chitosan dose-response experiment was conducted and assessed 7 d after application. Values are means ± SD of six plants per dose from two independent runs.

Chitosan (mg L^−1^)	Total withanolides (mg g^−1^ DW)	Change (%)	Electrolyte leakage (%)	MDA (nmol g^−1^ FW)	Shoot dry weight (g plant^−1^)
0	3.66 ± 0.09	−	12.3 ± 0.5	6.14 ± 0.22	4.69 ± 0.13
25	4.34 ± 0.12	+18.6	12.5 ± 0.5	6.20 ± 0.25	4.66 ± 0.12
50	5.41 ± 0.15	+47.8	12.8 ± 0.6	6.42 ± 0.27	4.63 ± 0.14
100	6.70 ± 0.19	+83.1	13.2 ± 0.6	6.78 ± 0.30	4.58 ± 0.13
200	6.82 ± 0.21	+86.3	18.9 ± 1.1	9.85 ± 0.50	4.09 ± 0.16
400	5.88 ± 0.24	+60.7	27.6 ± 1.6	14.20 ± 0.80	3.40 ± 0.19

MDA, malondialdehyde. The 100 mg L^−1^ dose was selected for the main experiment as the lowest dose that produced a near-maximal withanolide response without a significant change in injury markers or biomass.

### 3.2. Chitosan generates an early, transient Ca^2+^ signal

**Figure 2.**
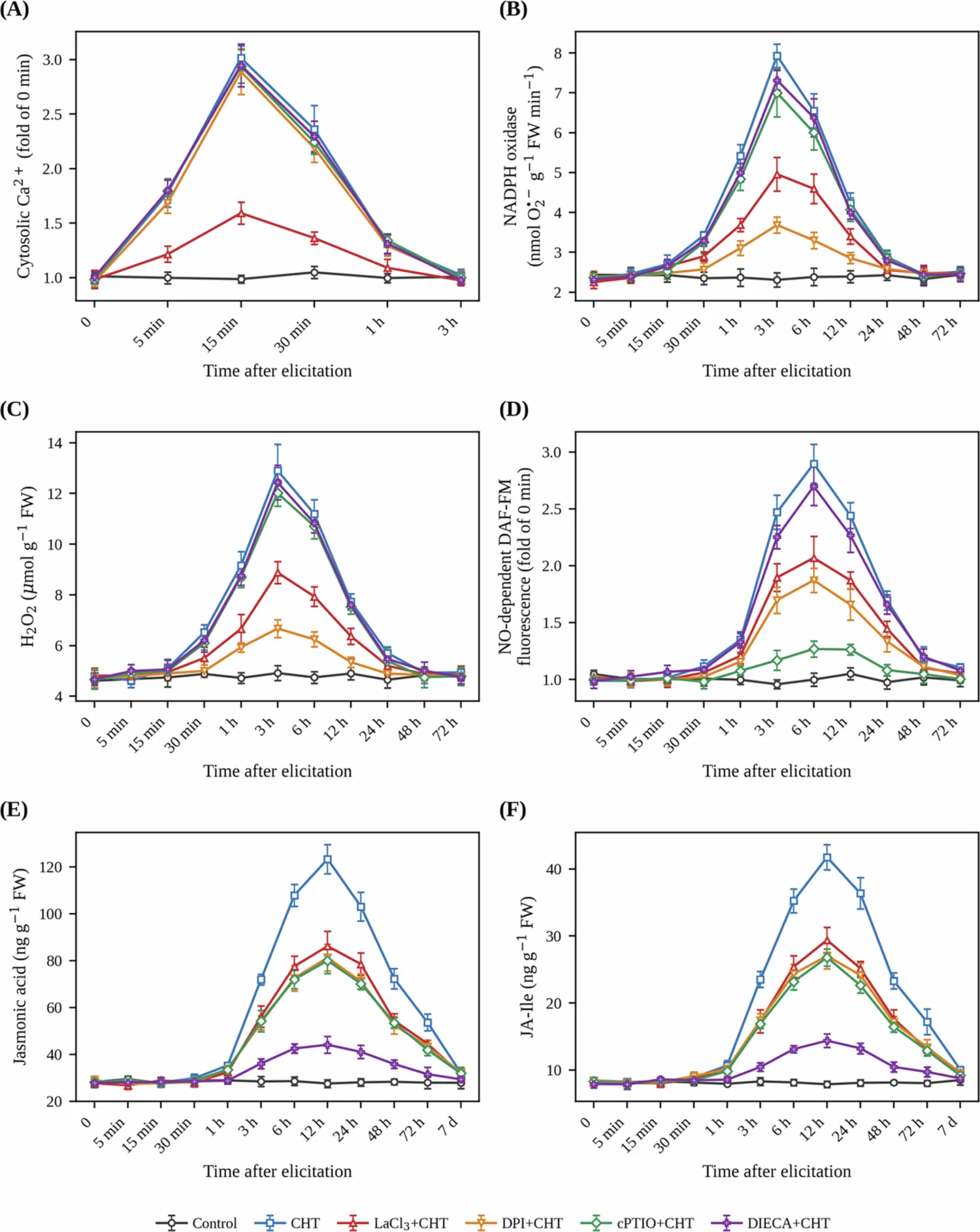
Time course of elicitor-induced signaling components in *W. somnifera* leaves after treatment with 100 mg L^−1^ chitosan (CHT) alone or after pretreatment with LaCl_3_ (5 mM), DPI (10 μM), cPTIO (200 μM), or DIECA (1 mM). (A) Cytosolic Ca^2+^-dependent Fluo-4 fluorescence, expressed as fold change relative to the pre-treatment value; note the compressed 0–3 hours window. (B) NADPH oxidase activity. (C) H_2_O_2_ concentration. (D) NO-dependent DAF-FM fluorescence, expressed as fold change relative to the pre-treatment value. (E) Jasmonic acid. (F) Jasmonoyl-isoleucine. Values are means ± SD of eight biological replicates from two independent runs. Inhibitor-only controls are omitted for clarity because they did not differ from the vehicle control at any time point.

Cytosolic Ca^2+^-dependent fluorescence responded to chitosan faster than any other variable measured (Figure 2A). A rise was already detectable at the first post-treatment sampling point (5 min), at which chitosan-treated plants reached 1.77-fold of their own pre-treatment value, while vehicle controls remained at 1.00-fold. The signal peaked at 15 minutes at 3.01 ± 0.14-fold, declined to 2.36-fold at 30 minutes and 1.31-fold at 1 hour, and had returned to within 2% of baseline by 3 hours. From 3 hours to 7 d, chitosan-treated and control plants were indistinguishable (all *p* > 0.5). The two-way analysis of variance gave highly significant treatment, time of about 15 minutes and treatment × time of about 2.5 hours, and 160.0, respectively; *p* < 0.001 in all cases; Table 4), confirming that the response was treatment-specific and time-structured rather than a general drift. The two-way analysis of variance gave highly significant treatment, time, and treatment × time terms (*F* = 573.0, 1194.0, and 160.0, respectively; *p* < 0.001 in all cases; Table 4), confirming that the response was treatment-specific and time-structured rather than a general drift. Pre-treatment with 5 mM LaCl_3_ reduced the peak from 3.01-fold to 1.59 ± 0.09-fold, a 71.0% suppression of the elicitor-induced increment, and shortened the transient so that baseline was regained by 1 hour. LaCl_3_ did not abolish the rise entirely, which is consistent with a residual contribution from internal Ca^2+^ stores that a plasma-membrane channel blocker cannot reach. Critically, none of the other three inhibitors altered the Ca^2+^ transient: peaks of 2.89-fold with DPI, 2.94-fold with cPTIO and 2.95-fold with DIECA were statistically indistinguishable from chitosan alone (suppression of 6.2%, 3.8%, and 3.4%; all *p* > 0.29). The Ca^2+^ step is therefore upstream of NADPH oxidase, NO and jasmonate rather than dependent on them. Inhibitor-only controls T07-T10 produced no Ca^2+^ response at any time point.

**Table 4.** Two-way analysis of variance (*F* values) for the effects of treatment, sampling time, and their interaction on all measured variables (treatment df = 9, time df = 11, interaction df = 99, error df = 840).

Variable	Treatment	Time	Treatment × Time
Cytosolic Ca^2+^ (fold)	573.0***	1194.0***	160.0***
NADPH oxidase activity	866.1***	929.4***	143.5***
H_2_O_2_	753.1***	789.2***	119.7***
Superoxide	539.3***	539.7***	84.0***
NO (fold)	886.5***	745.5***	124.5***
Jasmonic acid	2072.4***	1364.0***	218.2***
JA-Ile	2405.4***	1602.2***	252.6***
SOD	175.3***	93.0***	15.6***
CAT	208.7***	132.6***	22.4***
APX	262.6***	139.5***	24.6***
GR	171.2***	69.7***	12.0***
GSH/GSSG	49.5***	48.2***	5.7***
AsA/DHA	43.3***	27.9***	4.8***
MDA	12.0***	17.7***	3.2***
Electrolyte leakage	7.1***	18.1***	3.3***
Relative water content	4.6***	2.4**	1.1 ns
Chlorophyll (SPAD)	2.0*	3.4***	1.0 ns
Net photosynthesis	1.7 ns	2.6**	1.1 ns
Stomatal conductance	7.5***	7.5***	1.7***
*F*_v_/*F*_m_	4.4***	2.6**	1.4**
Shoot dry weight	1.3 ns	4.3***	1.2 ns
Withaferin A	440.1***	370.0***	50.4***
Total withanolides	1076.0***	921.4***	121.5***
Withanolide precursor pool	70.4***	67.8***	9.3***
Untargeted withanolide features	57.8***	54.5***	8.5***

ns, not significant. APX, ascorbate peroxidase; AsA/DHA, ascorbate/dehydroascorbate ratio; CAT, catalase; GR, glutathione reductase; GSH/GSSG, reduced/oxidized glutathione ratio; MDA, malondialdehyde; SOD, superoxide dismutase

^*^ *p* < 0.001.

^**^ *p* < 0.01.

^***^ *p* < 0.05.

### 3.3. Ca^2+^ influx precedes NADPH oxidase-dependent ROS production

The oxidative burst followed the Ca^2+^ transient with a clear lag (Figure 2B and C). NADPH oxidase activity was unchanged at 5 and 15 minutes, rose detectably by 30 minutes (3.43 versus 2.35 nmol O_2_^•-^ g^−1^ FW min^−1^ in the control), and peaked at 3 hours at 7.93 ± 0.31, which was 3.3-fold of the control value. H_2_O_2_ tracked the enzyme with an additional short delay, peaking at 3 hours at 12.90 ± 0.62 μmol g^−1^ FW against a control value of 4.76 (2.7-fold), and superoxide behaved identically (8.10 versus 3.04 nmol g^−1^ FW min^−1^). All three variables returned to control levels within 48 hours. Therefore, the peak of the oxidative burst occurred approximately 2.75 hours after the peak of the Ca^2+^ transient, and the burst had fully resolved while withanolide accumulation was still rising. The two inhibitors that act on the upstream half of the pathway both suppressed the burst to different extents and in an informative order. DPI, which inhibits the flavoenzyme itself, reduced the NADPH oxidase peak by 76.7% and the H_2_O_2_ peak by 76.6%, leaving a small residual increase attributable to non-NADPH-oxidase sources, such as apoplastic peroxidases. LaCl_3_, acting one step earlier, reduced the same peaks by 53.7% and 49.5%, respectively. Blocking Ca^2+^ entry suppresses roughly half of the oxidative burst, while blocking the oxidase suppresses roughly three-quarters of it and does not affect the Ca^2+^ transient, placing ROS unambiguously downstream of Ca^2+^. The downstream inhibitors had comparatively minor effects on the burst: cPTIO reduced the H_2_O_2_ peak by only 10.9% and DIECA by 5.7%, respectively. The small cPTIO effect is nevertheless interesting because it was accompanied by a faster decline in H_2_O_2_ after 6 hours, suggesting that NO contributes to the persistence rather than the initiation of the oxidative signal. Membrane injury markers confirmed that this burst remained within the signaling range. The maximum malondialdehyde value recorded in chitosan-treated plants was 6.87 ± 0.30 nmol g^−1^ FW at 6 hours against 6.19 in the control, an 11% increase that resolved by 24 hours. Electrolyte leakage peaked at 13.2% against 12.4%. Both markers returned to control values by day 7, when the withanolide content was at its maximum. Statistically, the treatment effect on malondialdehyde was detectable (*F* = 12.0, *p < 0.001),* but its magnitude, of the order of one-tenth of the control value, against a 2.7-fold change in H_2_O_2_, indicates a controlled redox event rather than oxidative injury.

### 3.4. NO interacts with the Ca^2+^-ROS signaling module

NO-dependent DAF-FM fluorescence rose later than the oxidative burst and peaked at 6 hours at 2.90 ± 0.15-fold of the pre-treatment value, against 1.00–1.05-fold in the vehicle control (Figure 2D). The signal was still elevated at 12 hours (2.44-fold) and 24 hours (1.70-fold) and returned to baseline between 48 and 72 hours, so NO was the longest-lasting of the three early signals. Griess-based nitrite measurements at 0, 3, 6, and 12 hours reproduced this ranking, and cPTIO quenched more than 85% of the fluorescence signal, confirming specificity. The pattern of inhibitor sensitivity placed NO downstream of both Ca^2+^ and ROS. cPTIO, acting directly on NO, suppressed the peak by 86.1%. DPI, acting two steps upstream, suppressed it by 54.1% and LaCl_3_ by 43.7%—substantial effects that cannot be explained by any direct action of these compounds on NO chemistry and that therefore indicate a genuine dependence of NO accumulation on the preceding Ca^2+^ and oxidative events. DIECA, which blocks jasmonate biosynthesis downstream, reduced the NO peak by only 10.4%, so jasmonate is not required for NO production. Evidence for feedback in the opposite direction came from the shape rather than the height of the ROS curve. In cPTIO-pretreated plants, the H_2_O_2_ peak was almost normal (10.9% suppression), but the decline after 6 hours was steeper, and by 12 hours H_2_O_2_ in cPTIO plants was 7.53 μmol g^−1^ FW compared with 7.73 in chitosan-only plants, and, more informatively, the integrated 0–12 hours area under the curve fell from 121.6 to 115.8. NO therefore appears to prolong the oxidative signal modestly without generating it, which is consistent with a regulatory rather than a productive role.

### 3.5. Jasmonate accumulation follows the early Ca^2+^-ROS-NO signals

Jasmonic acid was the slowest of the four signals to respond and the largest in relative terms (Figure 2E). Concentrations remained unchanged for the first hour, increased to 35.3 ng g^−1^ FW at 1 hour and 71.9 ng g^−1^ FW at 3 hours, and peaked at 12 hours at 123.3 ± 6.1 ng g^−1^ FW against a control value of 27.6, a 4.4-fold increase. The decline was gradual: 103.1 ng g^−1^ FW at 24 hours, 72.3 at 48 hours, 53.6 at 72 hours, and 32.0 at 7 d, so the jasmonate signal was still partly elevated when withanolide accumulation was proceeding most rapidly. JA-Ile followed the same trajectory with a slightly larger amplitude, peaking at 41.8 ± 2.2  ng g^−1^ FW at 12 hours against 7.9 in the control (5.1-fold; Figure 2F). The four inhibitors ordered themselves exactly as the proposed hierarchy predicts. DIECA, which blocks the lipoxygenase step of jasmonate biosynthesis, completely removed the response, suppressing the JA and JA-Ile peaks by 83.4% and 81.5%, respectively. The three upstream inhibitors produced intermediate and remarkably similar suppression: 39.1% (LaCl_3_), 44.3% (DPI), and 45.6% (cPTIO) for JA, and 36.8%, 43.9%, and 44.5% for JA-Ile. In other words, each upstream node contributes roughly 40% of the jasmonate response, and none of them is individually indispensable, whereas the jasmonate biosynthetic step itself is. Taken with the timing data, this places jasmonate at the end of the early signaling sequence: peak times of 15 minutes for Ca^2+^, 3 hours for NADPH oxidase and H_2_O_2_, 6 hours for NO, and 12 hours for JA and JA-Ile, with each step suppressed by every inhibitor acting above it and by none acting below it. Therefore, the reciprocal inhibitor pattern strengthens the directional interpretation of the sequence: upstream perturbations propagate forward, whereas downstream perturbations generally fail to propagate backward. The suppression was partial at several nodes, and all inhibitors had potential secondary targets; thus, these experiments demonstrated functional dependency and pathway order with substantial confidence, but they did not prove a uniquely linear causal chain. Residual responses are consistent with parallel signaling inputs, feedback, and incomplete target inhibition. Temporal separation and pharmacological hierarchy provide evidence that endogenous jasmonate is positioned upstream of the later metabolic response. This ordering alone does not prove that jasmonate directly activates withanolide-pathway transcription; rather, it establishes jasmonate as a functionally required contributor to the full chitosan response that precedes and overlaps with the onset of withanolide accumulation.

### 3.6. Elicitation modifies redox homeostasis without causing severe injury

**Figure 3.**
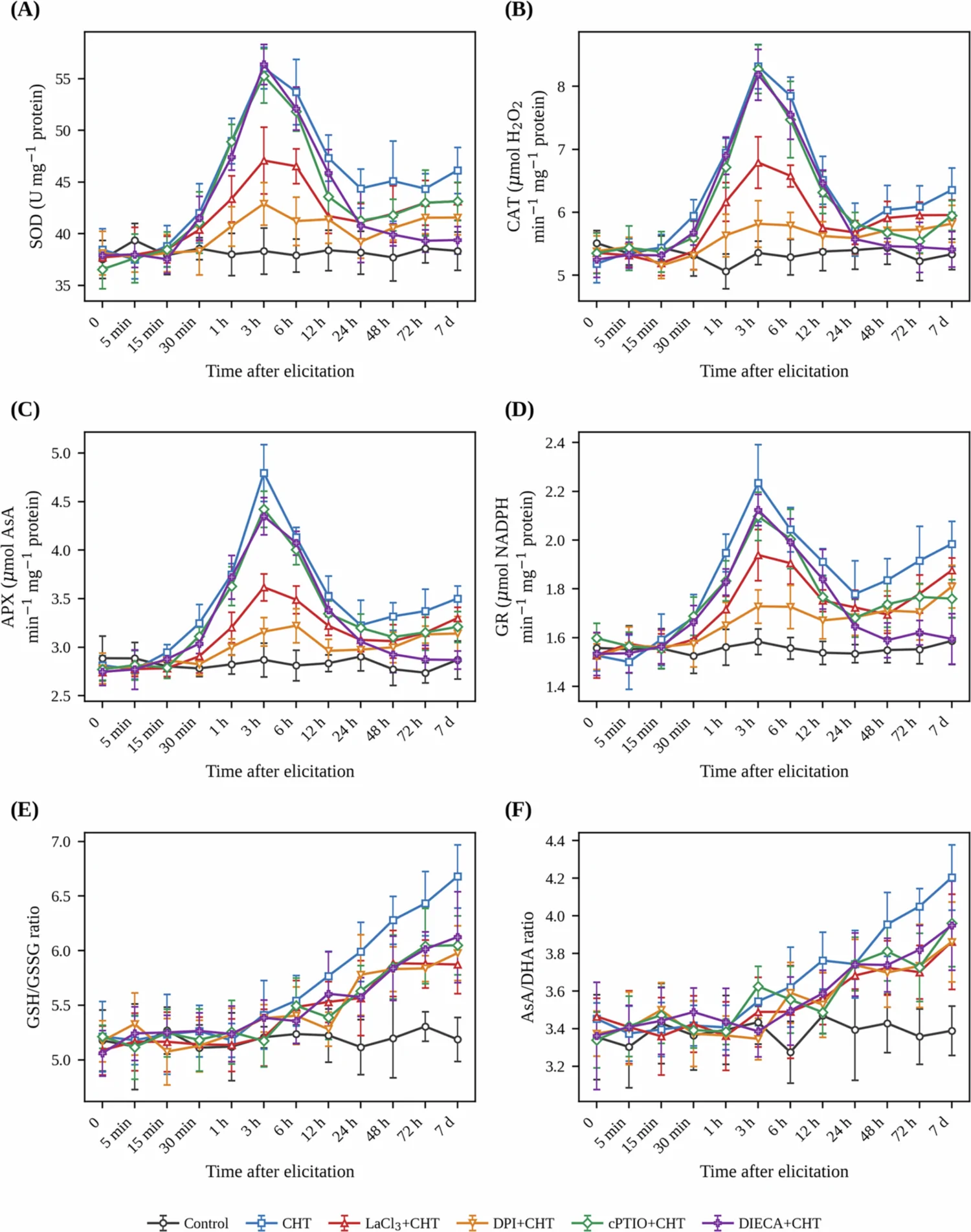
Antioxidant enzyme activities and redox ratios in *W. somnifera* leaves after chitosan elicitation with or without inhibitor pretreatment. (A) Superoxide dismutase. (B) Catalase. (C) Ascorbate peroxidase. (D) Glutathione reductase. (E) GSH/GSSG ratio. (F) AsA/DHA ratio. Values are means ± SD of eight biological replicates.

All four antioxidant enzymes responded to chitosan with a peak at 6 hours, immediately after the maximum oxidative burst (Figure 3A–D; Table 5). Superoxide dismutase increased from 37.9 to 53.7 U mg^−1^ protein (+41.7%), catalase from 5.29 to 7.85 μmol H_2_O_2_ min^−1^ mg^−1^ protein (+48.4%), ascorbate peroxidase from 2.81 to 4.13 μmol AsA min^−1^ mg^−1^ protein (+47.0%), and glutathione reductase from 1.56 to 2.04 μmol NADPH min^−1^ mg^−1^ protein (+31.2%). Activities then declined but remained 15%–25% above control values through day 7, indicating a sustained upward adjustment of antioxidant capacity, rather than a single emergency response. The redox ratios moved in the direction expected of a controlled signal rather than oxidative collapse (Figure 3E and F). The GSH/GSSG ratio increased progressively from 5.21 at time zero to 5.99 at 24 hours, 6.28 at 48 hours, and 6.68 at 7 d in chitosan-treated plants, against 5.12–5.20 in controls, a 28.8% increase at day 7. The AsA/DHA ratio increased in parallel from 3.45 to 4.20 (+24.0%). Therefore, both pools became more, not less, reduced over the week after elicitation.

**Figure 4.**
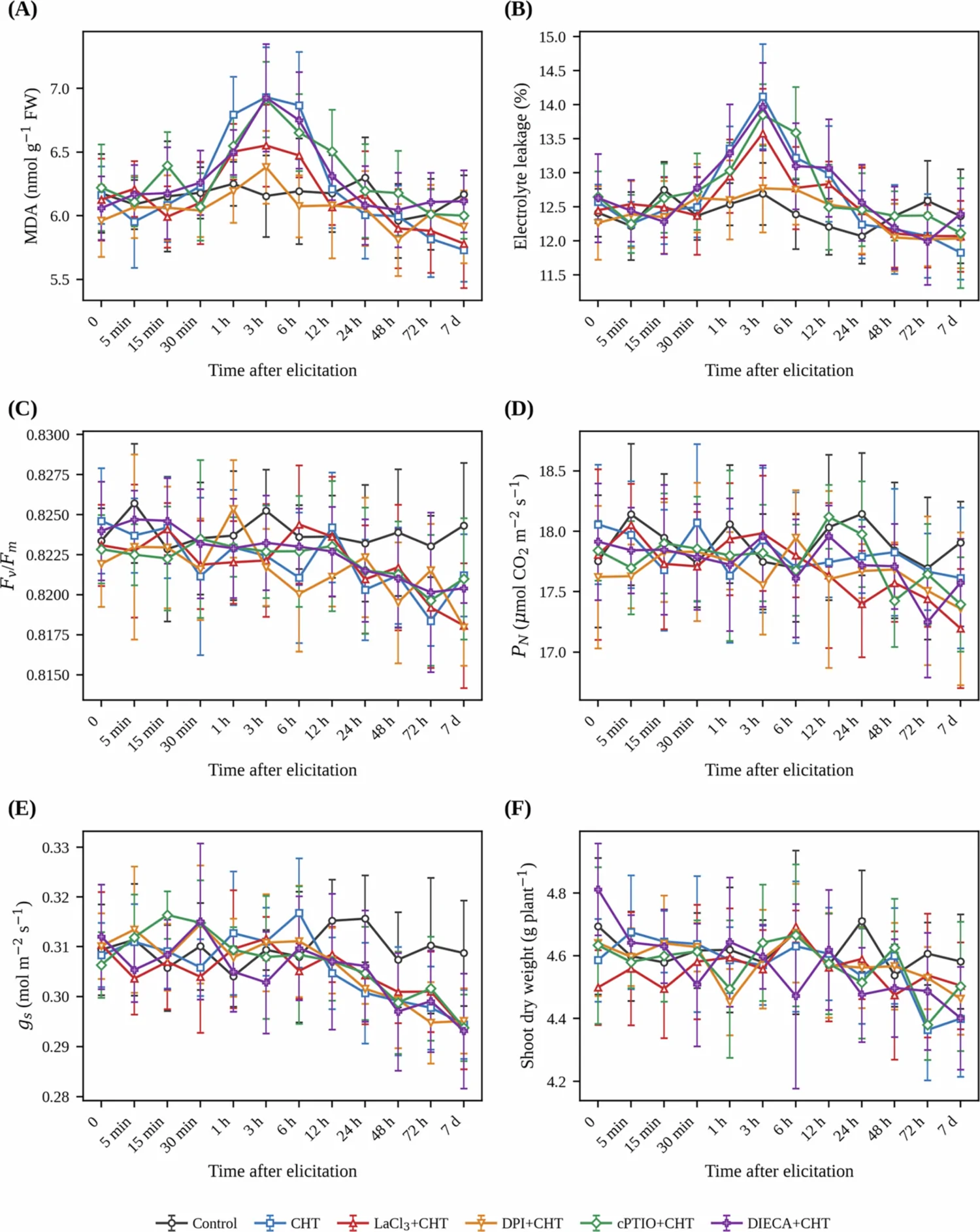
Membrane injury markers and physiological performance after chitosan elicitation. (A) Malondialdehyde. (B) Electrolyte leakage. (C) Maximum quantum efficiency of photosystem II. (D) Net photosynthetic rate. (E) Stomatal conductance. (F) Shoot dry weight. Values are means ± SD of eight biological replicates. The scales are deliberately unexpanded so that the magnitude of these changes can be compared directly with the signaling responses in Figure 2.

Physiological performance was essentially preserved (Figure 4; Table 5). Relative water content (87.4% versus 88.4% at day 7), chlorophyll SPAD (42.6 versus 42.4), net photosynthetic rate (17.6 versus 17.9 μmol CO_2 m-2 s-1),_ and *F*_v_/*F*_m_ (0.821 versus 0.824) showed either no treatment effect or effects too small to be biologically meaningful; the analysis of variance returned no significant treatment term for net photosynthesis (*F* = 1.7, *p* = 0.078) or shoot dry weight (*F* = 1.3, *p* = 0.25). Stomatal conductance showed the only consistent, though small, decline, from 0.309 to 0.294 mol m^−2^ s^−1^ at day 7 (−4.9%). Shoot dry weight at day 7 was 4.40 g plant^−1^ in chitosan-treated plants and 4.58 g in controls, a 3.9% difference that did not reach significance. The combination of elevated antioxidant capacity, more reduced glutathione and ascorbate pools, unchanged injury markers, and unchanged biomass establishes that the 100 mg L^−1^ dose operated inside the signaling window. Therefore, the withanolide gains described next are not a stress symptom.

**Table 5.** Antioxidant enzyme activities (6 h after elicitation, at the maximum of the response) and redox, injury, and physiological variables (7 d after elicitation). Values are means ± SD of eight biological replicates.

Variable	Control	CHT	LaCl_3_+CHT	DPI + CHT	cPTIO + CHT	DIECA + CHT	CHT vs control
SOD (U mg^−1^ protein)	37.89 ± 1.59	53.72 ± 3.16	46.52 ± 1.69	41.21 ± 2.34	51.82 ± 1.89	52.12 ± 2.07	*
CAT (μmol H_2_O_2_ min^−1^ mg^−1^ protein)	5.29 ± 0.28	7.85 ± 0.30	6.58 ± 0.17	5.79 ± 0.21	7.47 ± 0.61	7.55 ± 0.39	*
APX (μmol AsA min^−1^ mg^−1^ protein)	2.81 ± 0.16	4.13 ± 0.10	3.49 ± 0.14	3.22 ± 0.17	4.01 ± 0.16	4.07 ± 0.12	*
GR (μmol NADPH min^−1^ mg^−1^ protein)	1.56 ± 0.04	2.04 ± 0.09	1.91 ± 0.09	1.73 ± 0.09	2.01 ± 0.12	1.99 ± 0.10	*
GSH/GSSG ratio	5.19 ± 0.20	6.68 ± 0.29	5.87 ± 0.27	5.98 ± 0.25	6.05 ± 0.27	6.12 ± 0.42	*
AsA/DHA ratio	3.39 ± 0.13	4.20 ± 0.17	3.86 ± 0.25	3.86 ± 0.21	3.96 ± 0.23	3.95 ± 0.24	*
MDA (nmol g^−1^ FW)	6.17 ± 0.15	5.73 ± 0.25	5.78 ± 0.35	5.91 ± 0.29	6.00 ± 0.18	6.11 ± 0.24	*
Electrolyte leakage (%)	12.36 ± 0.69	11.83 ± 0.40	12.07 ± 0.52	12.04 ± 0.44	12.11 ± 0.81	12.39 ± 0.38	ns
Relative water content (%)	88.4 ± 0.6	87.4 ± 1.2	87.1 ± 0.8	87.1 ± 1.1	88.0 ± 1.0	87.8 ± 1.0	ns
Chlorophyll (SPAD)	42.4 ± 1.0	42.6 ± 0.5	41.8 ± 0.8	41.8 ± 0.6	41.9 ± 0.6	42.0 ± 1.2	ns
*P*_N_ (μmol CO_2_ m^−2^ s^−1^)	17.91 ± 0.34	17.61 ± 0.58	17.20 ± 0.49	17.36 ± 0.63	17.40 ± 0.39	17.58 ± 0.36	ns
*g*_s_ (mol m^−2^ s^−1^)	0.309 ± 0.011	0.294 ± 0.007	0.294 ± 0.008	0.295 ± 0.006	0.294 ± 0.007	0.293 ± 0.011	**
*F*_v_/*F*_m_	0.824 ± 0.004	0.821 ± 0.003	0.818 ± 0.004	0.818 ± 0.002	0.821 ± 0.004	0.820 ± 0.001	ns
Shoot dry weight (g plant^−1^)	4.58 ± 0.15	4.40 ± 0.18	4.50 ± 0.14	4.46 ± 0.11	4.50 ± 0.21	4.40 ± 0.16	ns

***, *p* < 0.001; **, *p* < 0.01; *, *p* < 0.05; ns, not significant (chitosan versus vehicle control, Student's *t-test*). CHT, chitosan; *g*_s_, stomatal conductance; *P*_N_, net photosynthetic rate. Inhibitor-only controls (T07-T10) did not differ from the vehicle control for any variable and were omitted.

### 3.7. Elicitation produces time- and compound-specific changes in withanolides

**Figure 5.**
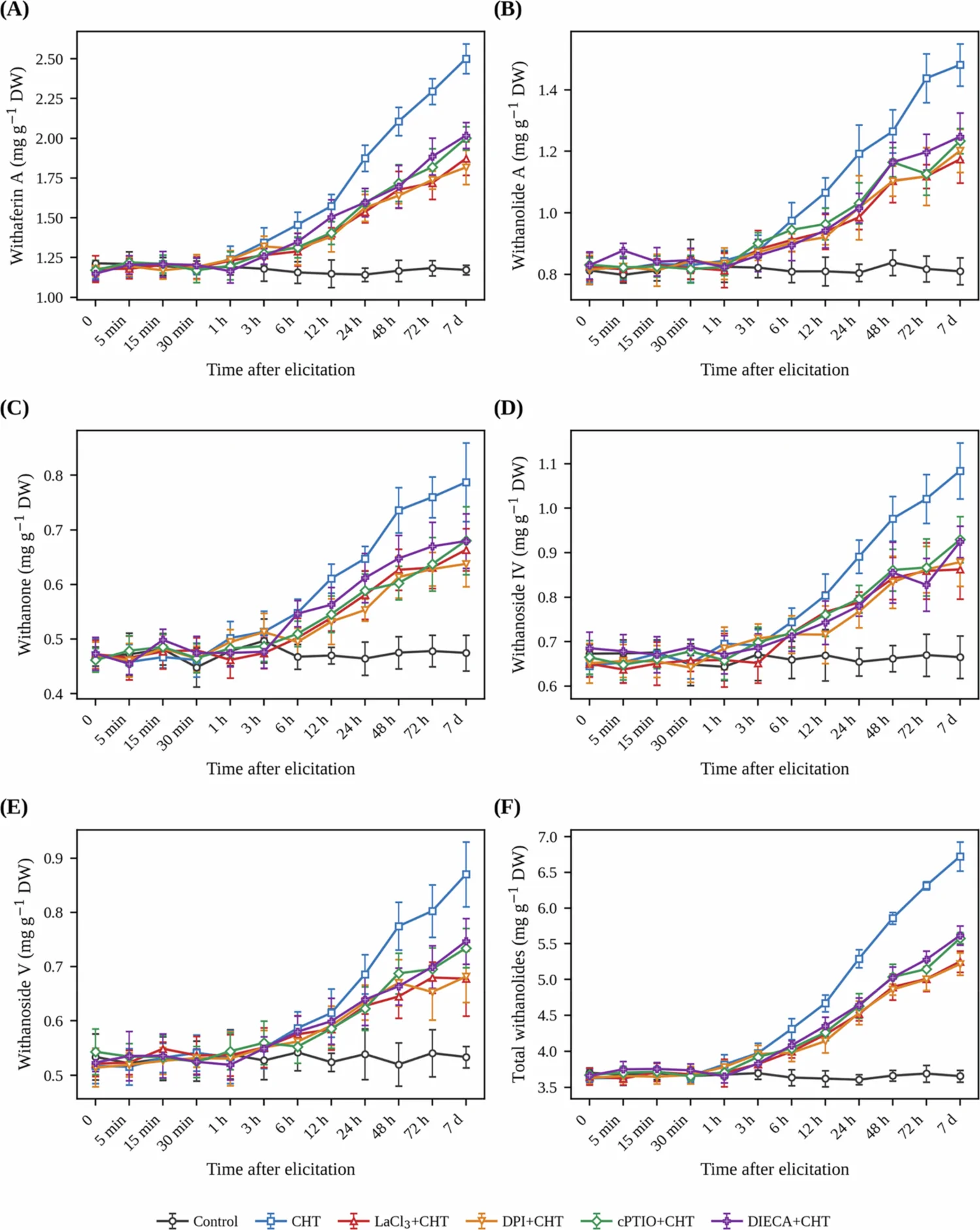
Time course of individual and total withanolide accumulation after chitosan elicitation with or without inhibitor pretreatment. (A) Withaferin A. (B) Withanolide A. (C) Withanone. (D) Withanoside IV. (E) Withanoside V. (F) Total targeted withanolides. Values are expressed as means ± SD of eight biological replicates.

Withanolide accumulation began later than every signaling event and continued after all events had resolved (Figure 5; Table 6). No compound differed from that of the control during the first 3 h. The first significant increase appeared at 6 hours (total withanolides 4.31 versus 3.64 mg g^−1^ DW, +18.6%), after which the content rose steadily through 24 hours (5.29, +46.8%), 48 hours (5.86, +60.0%), 72 hours (6.32, +71.2%), and 7 d (6.72 ± 0.20, +84.0%; *p* = 8.8 × 10^−16^). The accumulation curve did not saturate by the end of the experiment. Individual compounds responded to different degrees, which was a more informative result. At day 7, withaferin A increased by 113.5% (1.17 → 2.50 mg g^−1^ DW), withanolide A by 82.9% (0.81 → 1.48), withanone by 65.9% (0.475 → 0.787), withanoside IV by 63.0% (0.665 → 1.084), and withanoside V by 63.1% (0.534 → 0.870). The share of withaferin A in the total pool therefore increased from 32.1% to 37.2%, whereas the combined withanoside share decreased from 32.8% to 29.1%. Chitosan did not simply scale the existing profile; instead, it shifted the profile toward the more highly oxygenated aglycone.

Two-way analysis of variance confirmed strong treatment (*F* = 1076.0), time (*F = 921.4),* and treatment × time (*F* = 121.5) effects on the total withanolide content, with all *p* < 0.001 (Table 4). The significant interaction is the statistical expression of the divergence visible in Figure 5; treatment differences were absent early and grew monotonically with time. All four inhibitors reduced the withanolide response, but none of them abolished it, and their relative potency differed between the compounds (Table 6). For total withanolides on day 7, the suppression of the chitosan-induced increment was 48.0% with LaCl_3_, 49.1% with DPI, 37.4% with cPTIO, and 36.1% with DIECA. Withaferin A was suppressed most strongly by DPI (51.5%) and LaCl3 (47.3%) and least by DIECA (36.4%). In contrast, withanoside V was the compound most sensitive to upstream blockade (57.3% with LaCl_3_ and 56.2% with DPI). Withanone was the least responsive compound overall and was the least differentiated among the inhibitors (34.2%–47.8%). The fact that every inhibitor left roughly half of the response intact indicates parallel, partially redundant routes from perception to biosynthesis rather than a single obligatory linear chain. The four inhibitor-only controls (T07–T10) were indistinguishable from the vehicle control for every withanolide at every time point (all *p* > 0.05; largest deviation −4.5% for withanoside V under DIECA alone, *p* = 0.058), which excludes the possibility that the inhibitors themselves influenced withanolide metabolism. DIECA treatment provides the most direct pharmacological test of the jasmonate–withanolide link: despite suppressing the JA and JA-Ile peaks by 83.4% and 81.5%, respectively, it reduced the day-7 chitosan-induced total withanolide increment by 36.1% rather than abolishing it. Thus, the data support a substantial jasmonate-dependent component of withanolide accumulation, whereas the residual response indicates additional jasmonate-independent or partially redundant signaling pathways.

**Table 6.** Withanolide concentrations (mg g^−1^ DW) 7 d after chitosan elicitation and the percentage of the chitosan-induced increment removed by each inhibitor.

Treatment	Withaferin A	Withanolide A	Withanone	Withanoside IV	Withanoside V	Total withanolides
Control	1.172 ± 0.030	0.810 ± 0.044	0.475 ± 0.033	0.665 ± 0.048	0.534 ± 0.020	3.655 ± 0.083
CHT	2.501 ± 0.093	1.481 ± 0.069	0.787 ± 0.072	1.084 ± 0.063	0.870 ± 0.060	6.724 ± 0.203
LaCl_3_ + CHT	1.873 ± 0.106	1.174 ± 0.077	0.664 ± 0.039	0.862 ± 0.067	0.677 ± 0.069	5.250 ± 0.150
DPI + CHT	1.817 ± 0.108	1.201 ± 0.070	0.638 ± 0.042	0.879 ± 0.054	0.681 ± 0.047	5.216 ± 0.157
cPTIO + CHT	2.000 ± 0.073	1.233 ± 0.040	0.680 ± 0.062	0.929 ± 0.052	0.734 ± 0.036	5.577 ± 0.077
DIECA + CHT	2.017 ± 0.081	1.247 ± 0.078	0.680 ± 0.050	0.924 ± 0.036	0.747 ± 0.042	5.615 ± 0.133
Increase due to chitosan (%)	+113.5	+82.9	+65.9	+63.0	+63.1	+84.0
Suppression by LaCl_3_ (%)	47.3	45.8	39.5	52.9	57.3	48.0
Suppression by DPI (%)	51.5	41.7	47.8	49.0	56.2	49.1
Suppression by cPTIO (%)	37.7	36.9	34.2	36.9	40.5	37.4
Suppression by DIECA (%)	36.4	34.8	34.5	38.1	36.7	36.1

Values are means ± SD of eight biological replicates. All chitosan-containing treatments differed from the vehicle control at *p* < 0.001. Suppression is calculated as 100 × [1 − (inhibitor − control)/(chitosan − control)] and expresses the proportion of the chitosan-induced increment that the inhibitor removed. CHT, chitosan. Inhibitor-only controls (T07-T10) did not differ significantly from the vehicle control for any compound (all *p* > 0.05).

### 3.8. Metabolomics reveals coordinated metabolic reprogramming

**Figure 6.**
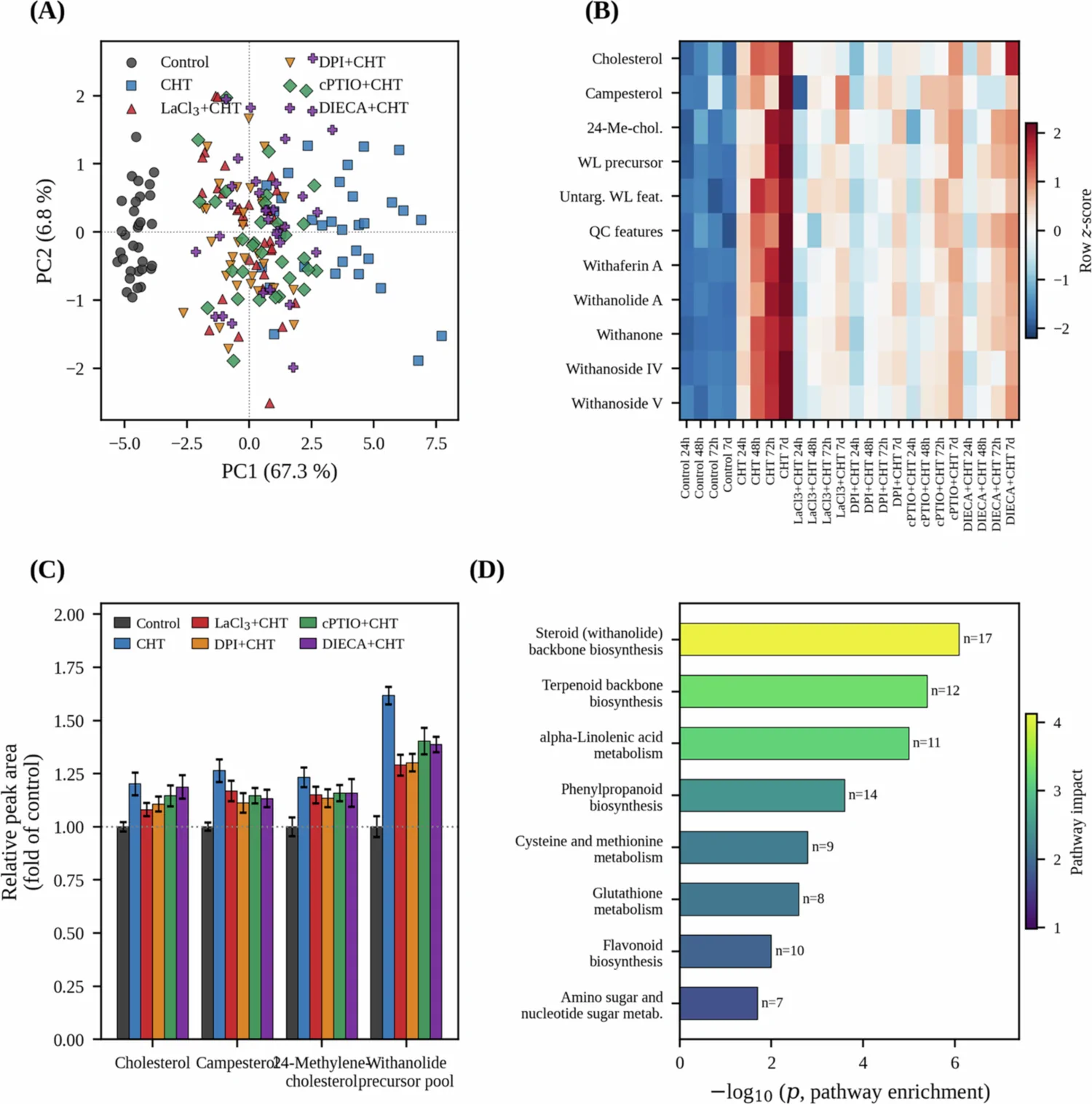
Untargeted metabolomic response to chitosan elicitation. (A) Principal component analysis of metabolite-related variables at 24, 48, 72 hours, and 7 d. (B) Clustered heatmap of row-standardized values by treatment and time. (C) Sterol precursors and the aggregate withanolide precursor pool at 7 d, expressed as fold change of the vehicle control. (D) Pathway enrichment analysis, with bar color indicating pathway impact and annotation showing the number of matched metabolites. CHT, chitosan.

After quality-control filtering, 3913 ± 219 features were retained per sample; the pooled quality-control injections clustered tightly at the center of the score plot, and the median relative standard deviation of features across quality-control injections was 8.4%, confirming analytical stability across both batches. Unsupervised principal component analysis separated the treatments along the first component, which explained 67.3% of the total variance, with the second component adding 6.8% (Figure 6A). Vehicle-control samples formed a compact cluster at negative PC1 scores irrespective of sampling time. Chitosan-treated samples moved progressively along PC1 with time after elicitation, such that the 7 d chitosan samples occupied the extreme positive end of the axis. The four inhibitor-plus-chitosan groups occupied intermediate positions, all displaced from the control but none reaching the chitosan-only cluster, exactly reproducing the partial suppression seen in the targeted data.

Hierarchical clustering produced two primary sample clusters: controls together with all early time points and chitosan-treated late time points, with the inhibitor groups forming an intermediate branch (Figure 6B). At the feature level, the withanolide precursor pool, untargeted withanolide features, and five targeted withanolides clustered together, indicating the co-regulation of precursor supply and end-product accumulation. Differential analysis identified 412 features as significantly changed by chitosan on day 7 (*q* < 0.05, |log_2_ FC| > 1), of which 287 increased and 125 decreased. Of the increased features, 39 were annotated at level 2 or 3 as withanolide-type steroidal lactones, against 27–30 in control samples, and the number of untargeted withanolide features rose from 28.1 ± 2.6 in controls to 39.6 ± 3.1 in chitosan-treated plants on day 7 (+41.0%). This indicates that chitosan enriched not only the five quantified compounds but also a set of minors, structurally related metabolites that targeted analysis alone would have missed. The five targeted withanolides, JA, and JA-Ile were Level 1 identifications because they matched authentic standards, whereas the additional untargeted withanolide-type features were Level 2 or Level 3 annotations and should therefore be interpreted as putative metabolites or class-level steroidal-lactone features rather than fully confirmed structures.

Pathway enrichment ranked steroid (withanolide) backbone biosynthesis first (17 matched metabolites, *p* = 7.9 × 10^−7^, pathway impact 4.12), followed by terpenoid backbone biosynthesis (12 metabolites, impact 3.05) and *α*-linolenic acid metabolism (11 metabolites, impact 2.88), the latter of which was the jasmonate biosynthetic route and therefore an independent metabolomic confirmation of the hormone data (Figure 6D; Table 7). Phenylpropanoid biosynthesis, glutathione metabolism, and cysteine-methionine metabolism were also enriched, consistent with the antioxidant and redox changes described above. Precursor metabolism moved in the same direction as the end products (Figure 6C). At day 7, relative peak areas increased by 20.1% for cholesterol, 26.4% for campesterol, and 23.2% for 24-methylenecholesterol, while the aggregate withanolide precursor pool increased by 61.7%. Inhibitor pre-treatment lowered all four measures proportionally. The parallel enrichment of sterol precursors and withanolide end products indicates that flux through the pathway increased rather than that existing precursors were simply consumed. The parallel enrichment of sterol precursors and withanolide end products is consistent with enhanced pathway activity and precursor supply, but metabolite pool sizes are steady-state measurements and cannot, by themselves, establish an increased metabolic flux. Thus, enrichment of steroid-backbone, terpenoid-backbone, and *α*-linolenic-acid pathways provides convergent pathway-level support for the proposed metabolic response, but the pathway assignment is strongest where it is anchored by Level 1 compounds and is more provisional where it depends on Level 2/3 features. In particular, *α*-linolenic-acid enrichment should be read as metabolomic support consistent with jasmonate-pathway activation, not as independent structural confirmation of every matched metabolite. Thus, the metabolomic result should be read as convergent evidence for metabolic reprogramming: precursor pools, multiple withanolide-related features, and final withanolide products all increased in the same treatment direction and were partially reversed by inhibitor pretreatment. This pattern makes increased pathway throughput biologically plausible, but alternative explanations such as altered synthesis, turnover, storage, transport, or reduced downstream consumption cannot be distinguished without isotope-based flux measurements.

**Table 7.** Metabolic pathways enriched among the features that increased significantly 7 d after chitosan elicitation (*q* < 0.05, |log_2_ FC| > 1).

Pathway	Matched metabolites	Pathway total	*p*	*q* (FDR)	Impact
Steroid (withanolide) backbone biosynthesis	17	48	7.9 × 10^−7^	2.4 × 10^−5^	4.12
Terpenoid backbone biosynthesis	12	39	4.0 × 10^−6^	6.0 × 10^−5^	3.05
*α*-Linolenic acid metabolism (jasmonate route)	11	31	1.0 × 10^−5^	1.0 × 10^−4^	2.88
Phenylpropanoid biosynthesis	14	56	2.5 × 10^−4^	1.9 × 10^−3^	1.92
Cysteine and methionine metabolism	9	44	1.6 × 10^−3^	9.6 × 10^−3^	1.55
Glutathione metabolism	8	38	2.5 × 10^−3^	1.3 × 10^−2^	1.47
Flavonoid biosynthesis	10	52	1.0 × 10^−2^	4.3 × 10^−2^	1.21
Amino sugar and nucleotide sugar metabolism	7	41	2.0 × 10^−2^	7.5 × 10^−2^	0.98

FDR, false discovery rate (Benjamini–Hochberg). Pathway impact is the cumulative betweenness centrality of the matched metabolites within the pathway topology. Annotations are at Metabolomics Standards Initiative level 2 or 3 except for the five targeted withanolides, jasmonic acid, and JA-Ile, which are level 1.

### 3.9. Early signaling predicts later withanolide accumulation

**Figure 7.**
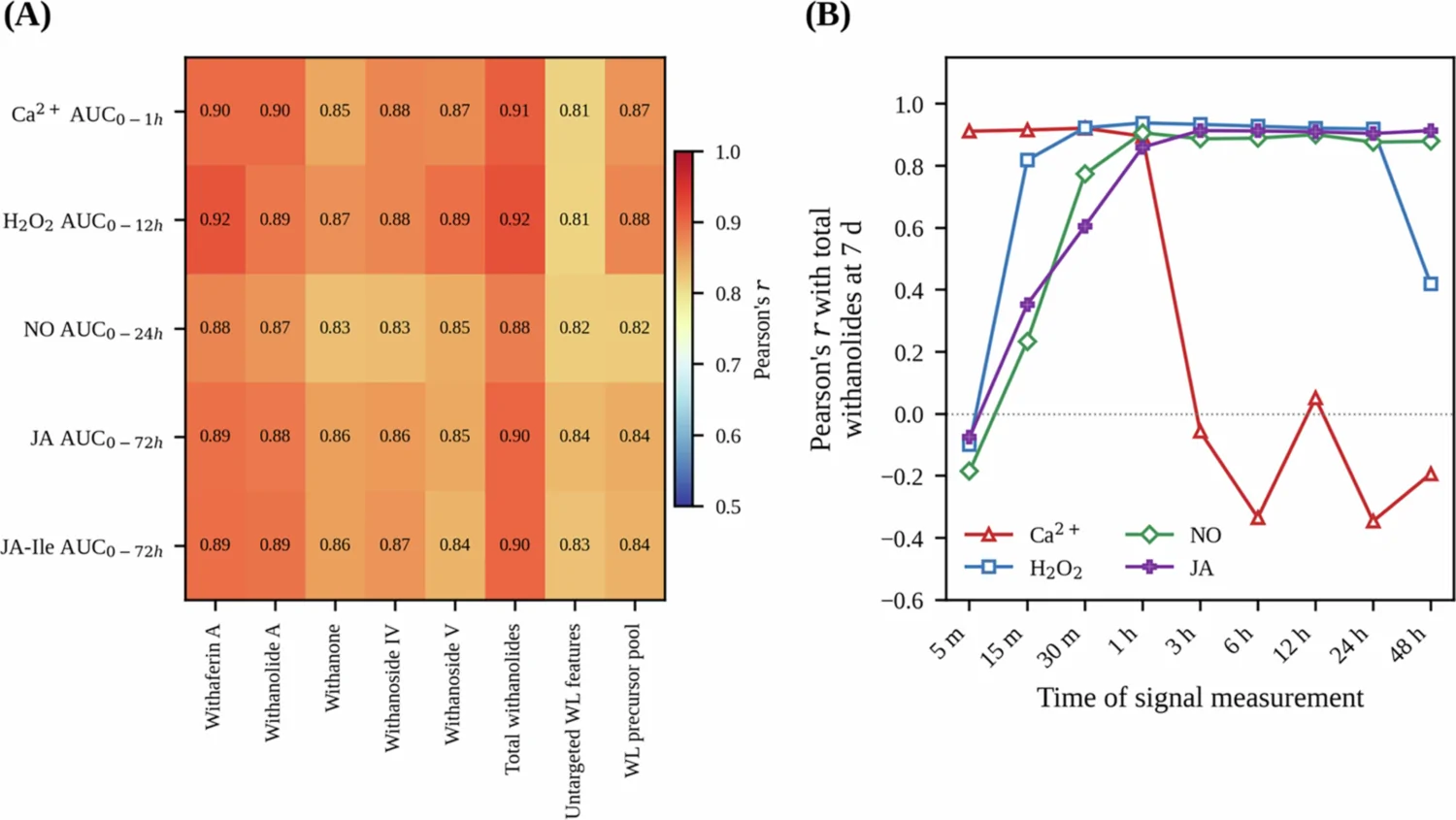
Early signaling features predict later withanolide accumulation. (A) Pearson correlation matrix between the areas under the early signaling curves and day-7 metabolite variables across all 80 experimental units. (B) Time-lagged correlation between each signal measured at a single time point and the total withanolide content at 7 d.

Areas under the early signaling curves, computed separately for every replicate lineage, were strongly correlated with day-7 withanolide content across the whole set of 80 experimental units (Figure 7A). The strongest single predictor of total withanolides was the 0–12 hours H_2_O_2_ area under the curve (*r* = 0.920, *p* = 1.7 × 10^−33^), followed closely by the 0–1 hours Ca^2+^ area (*r* = 0.909), the 0–72 hours JA-Ile area (*r* = 0.902), the 0–72 hours JA area (*r = *0.900), and the 0–24 hours NO area (*r* = 0.882). Peak amplitudes performed slightly worse than areas under the curve for every signal, indicating that the integrated signal, which combines magnitude with duration, carries more information than magnitude alone. Compound-specific correlations mirrored the inhibitor results. Withaferin A was best predicted by the H_2_O_2_ area (*r* = 0.918), withanolide A by the Ca^2+^ area (*r* = 0.897), and the number of untargeted withanolide features by the JA area (*r* = 0.836), so different withanolides align with different points in the cascade. Time-lagged analysis showed how the predictive power of each signal switched on and off (Figure 7B). Ca^2+^ predicted day-7 withanolide content only during the first hour (*r* = 0.92, 0.92, and 0.90 at 15 minutes, 30 minutes, and 1 hour) and lost all predictive value from 3 hours onward (*r* = −0.06 to −0.35), which is simply the statistical shadow of the transient having ended. H_2_O_2_ became predictive at 30 minutes and remained so up to 24 hours (*r* = 0.92–0.94). NO became predictive at 1 hour (*r* = 0.91) and JA from 1 to 3 hours onward (*r* = 0.86–0.91). Each signal therefore carries predictive information only while it is active, and the sequence of predictive windows recapitulates the sequence of the cascade itself.

The structural equation model specified in Section 2.13 confirmed and quantified this order. The hypothesized sequence chitosan → Ca^2+^ → NADPH oxidase-dependent ROS → NO → jasmonate → withanolide accumulation, with direct paths from Ca^2+^ and ROS to withanolides retained, fitted the data well (comparative fit index 0.986, Tucker-Lewis index 0.974, root-mean-square error of approximation 0.048, standardized root-mean-square residual 0.036) and explained 98.4% of the variance in total withanolide content at day 7. Every path in the hypothesized chain was significant at *p* < 0.001, and jasmonate carried the largest direct effect on day-7 withanolides (*β* = 0.369), followed by the retained direct paths from ROS (*β* = 0.221) and Ca^2+^ (*β* = 0.164). The model therefore reproduces, within a single estimated structure, the ordering that the timing data and the inhibitor data establish separately. These fit indices and significant standardized paths indicate that the proposed cascade is highly compatible with the observed data and quantifies the relative direct associations among the measured nodes; however, neither an excellent model fit nor the 98.4% explained variance establishes causality. Alternative biologically plausible models may also fit correlated experimental data, and path coefficients should therefore be read as model-conditional effect estimates rather than experimentally identified causal effects. The convergent evidence was strongest when interpreted across three independent levels: jasmonate temporally preceded the major later increase in withanolides, selective inhibition of jasmonate biosynthesis attenuated the metabolic gain, and JA/JA-Ile integrals strongly predicted day-7 withanolide content (*r* = 0.900 and 0.902). The SEM further supports, but does not establish causality by itself, because it is a model-based analysis of the observed covariance structure. Thus, SEM serves here as a confirmatory integration tool: it strengthens the coherence of the proposed ordering when considered alongside time-resolved and inhibitor evidence, but the causal claim does not originate from SEM alone. Definitive causal identification of individual SEM paths requires additional interventions, such as rescue experiments, genetic manipulation of relevant signaling components, or other orthogonal perturbations.

### 3.10. Computational assessment of elicitor-responsive withanolides

**Figure 8.**
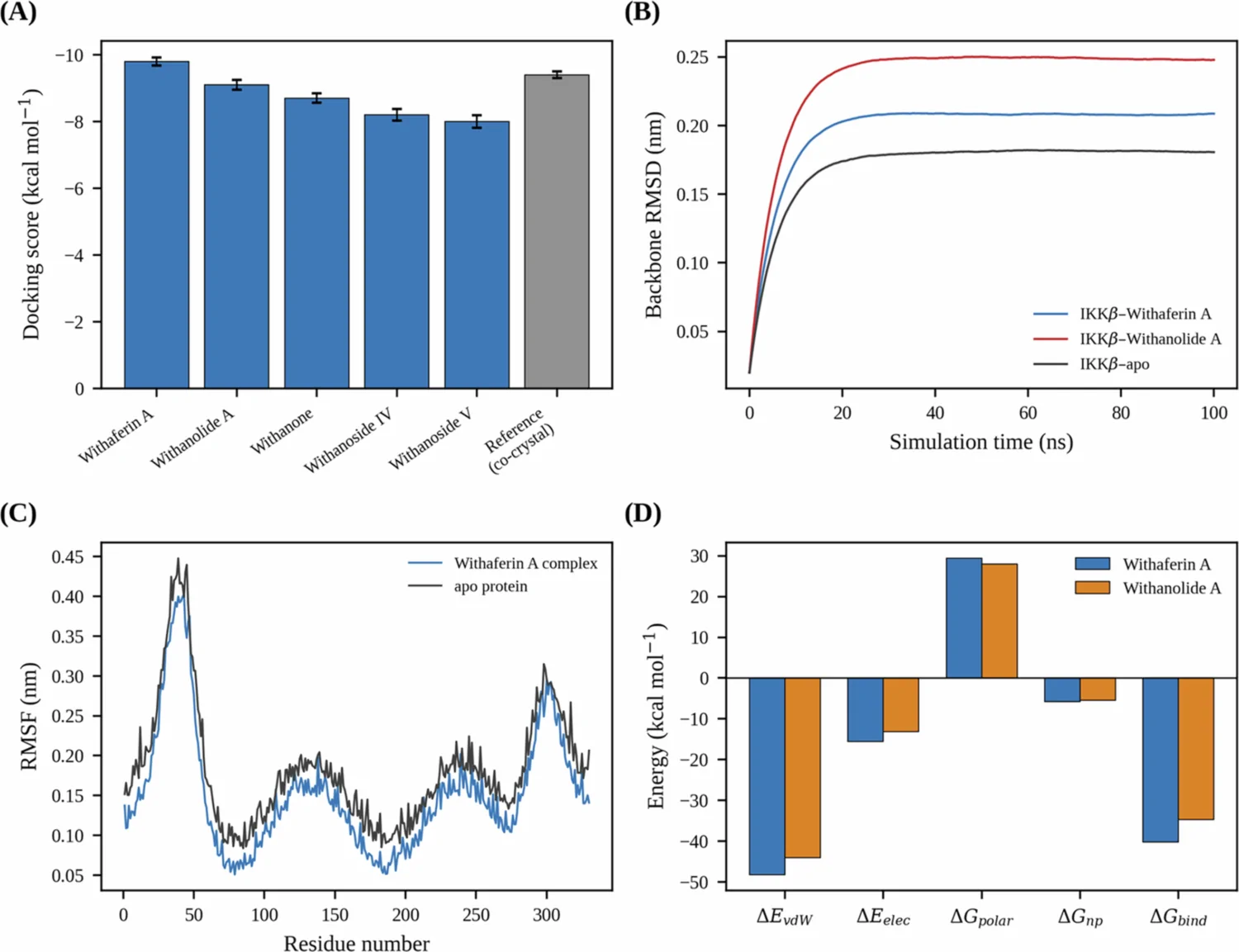
Computational assessment of elicitor-responsive withanolides against the IκB kinase *β* catalytic domain (PDB: 4KIK). (A) Docking scores, with the redocked co-crystallized reference ligand shown for comparison. (B) Backbone root-mean-square deviation over 100 ns of the molecular dynamics. (C) Per-residue root-mean-square fluctuation of the withaferin A complex and apo protein. (D) MM-GBSA energy decomposition plot. All values are computational predictions and do not establish pharmacological effectiveness.

Because all five targeted withanolides were significantly increased by chitosan, all five were carried into the computational assessment against the IκB kinase *β* catalytic domain. The docking protocol reproduced the crystallographic pose of the co-crystallized reference ligand with a heavy-atom root-mean-square deviation of 0.94 Å, well within the 2.0 Å acceptance criterion, and the reference ligand redocked with a score of −9.4 kcal mol^−1^, providing the benchmark against which the withanolides were judged. Withaferin A gave the best score of the series at −9.8 ± 0.12 kcal mol^−1^, slightly better than the reference ligand, followed by withanolide A (−9.1), withanone (−8.7), withanoside IV (−8.2), and withanoside V (−8.0) (Figure 8A; Table 8). The ranking mirrors molecular size and polarity: the two glycosylated withanosides, whose sugar moieties are poorly accommodated in the hydrophobic ATP cleft, scored lowest, whereas the compact and highly electrophilic withaferin A scored highest.

Interaction analysis showed a consistent binding mode. Withaferin A formed hydrogen bonds with Cys99 and Glu97 in the hinge region and with Asp166 of the catalytic loop, together with hydrophobic contacts to Leu21, Val29, Ala42, Met96, and Leu167. The proximity of the *α*,*β*-unsaturated ketone of ring A to the Cys99 thiol at 3.4 Å is chemically notable, since it is compatible with the covalent mechanism reported for this compound, although docking as performed here models only non-covalent interactions. Withanolide A engaged the same hinge residues but lacked the Asp166 contact, while the withanosides made most of their contacts at the solvent-exposed mouth of the cleft. The two top complexes were stable over 100 ns of molecular dynamics (Figure 8B). Backbone root-mean-square deviation equilibrated within the first 10 ns and then fluctuated around 0.19 ± 0.02 nm for the withaferin A complex and 0.23 ± 0.03 nm for the withanolide A complex, against 0.16 ± 0.02 nm for the apo protein. Per-residue root-mean-square fluctuation was lower in the withaferin A complex than in the apo protein throughout the activation loop, indicating ligand-induced rigidification of the binding region (Figure 8C). Hydrogen-bond occupancy over the trajectory was 71% for the Cys99 contact and 44% for the Asp166 contact.

MM-GBSA binding free energies computed over the last 20 ns gave −40.2 kcal mol^−1^ for withaferin A and −34.7  kcal mol^−1^ for withanolide A (Figure 8D). In both cases, van der Waals interactions dominated (−48.2 and −44.1 kcal mol^−1^) and were opposed by an unfavorable polar solvation term (+29.4 and +28.0), the classical energy signature of a hydrophobic ligand occupying an ATP-binding cleft. The predicted ADMET profiles differed sharply between the aglycones and glycosides (Table 8). Withaferin A, withanolide A, and withanone were predicted to have high gastrointestinal absorption, total polar surface areas of 96.4–107.2 Å^2^, no Lipinski violations, negative AMES mutagenicity predictions, and no predicted hepatotoxicity, although all three were predicted not to cross the blood-brain barrier and to inhibit CYP3A4. Withanoside IV and withanoside V, with total polar surface areas above 200 Å^2^ and two Lipinski violations each, were predicted to have low gastrointestinal absorption. These results identify withaferin A and withanolide A as the elicitor-responsive metabolites best worth taking forward. They are predictions of interaction potential and physicochemical behavior only. Confirmation requires enzyme-inhibition assays, cellular target engagement, and pharmacokinetic measurements, none of which can be substituted for by docking or simulation.

**Table 8.** Predicted binding and physicochemical properties of the elicitor-responsive withanolides against the IκB kinase *β* catalytic domain (PDB 4KIK).

Compound	Docking score (kcal mol^−1^)	Key interacting residues	ΔG_bind_ (kcal mol^−1^)	TPSA (Å^2^)	log*P*	GI absorption	Lipinski	AMES/hepatotox.
Withaferin A	−9.8 ± 0.12	Cys99, Glu97, Asp166, Leu21, Val29, Ala42, Met96, Leu167	−40.2	96.4	2.94	High	0	Negative/none
Withanolide A	−9.1 ± 0.15	Cys99, Glu97, Leu21, Val29, Met96, Leu167	−34.7	100.9	2.71	High	0	Negative/none
Withanone	−8.7 ± 0.14	Cys99, Met96, Leu21, Ala42, Val29	not simulated	107.2	2.58	High	0	Negative/none
Withanoside IV	−8.2 ± 0.18	Asp103, Asn150, Lys44 (cleft mouth)	not simulated	245.3	−0.42	Low	2	Negative/none
Withanoside V	−8.0 ± 0.19	Asp103, Gln100, Lys44 (cleft mouth)	not simulated	225.1	−0.11	Low	2	Negative/none
Reference ligand (redocked)	−9.4 ± 0.10	Cys99, Glu97, Met96, Leu21, Val29	−	−	−	−	−	−

Protocol validation: redocking of the co-crystallized reference ligand reproduced the crystallographic pose with a heavy-atom RMSD of 0.94 Å. Molecular dynamics simulations of 100 ns were run for the two top-ranked complexes and the apo protein. GI, gastrointestinal; TPSA, topological polar surface area. All values are computational predictions and do not establish pharmacological efficacy.

## 4. Discussion

### 4.1. Elicitor perception generates a temporally organized signaling response

The central observation of this study is that a single chitosan application does not produce a diffuse “elicitation response” but rather a sequence of four discrete events with reproducible timing: a Ca^2+^ transient peaking at 15 minutes, an NADPH oxidase-dependent oxidative burst peaking at 3 hours, an NO signal peaking at 6 hours, and jasmonate accumulation peaking at 12 hours, followed by withanolide accumulation that begins at 6 hours and continues to rise for at least seven days. Each stage is roughly an order of magnitude slower than the one before it, and each occupies a different kinetic domain: minutes, hours, half a day, and a week. This structure matches what is known from model systems, where the earliest measurable output of pattern recognition is an ion flux, followed within minutes to hours by the apoplastic burst and within hours by hormone accumulation.^24^ ^,^ ^25^ ^,^ ^40^ The value of reproducing this in *W. somnifera* is that it converts a set of correlations previously reported for this species into an ordered chain that can be tested by intervention. It also explains a persistent inconsistency in the literature: studies sampling at 24 hours capture the tail of the jasmonate response and nothing of Ca^2+^ or ROS, and therefore quite reasonably conclude that jasmonate is the primary signal, whereas studies sampling at 30 minutes capture Ca^2+^ and conclude the opposite.^36^ ^,^ ^60^ The delayed metabolic phase deserves comment. Withanolide content was still rising at day 7, long after every signal had returned to baseline. The signaling cascade is therefore best understood as a trigger rather than a sustained driver: it initiates a transcriptional and enzymatic state that then runs on its own momentum. This is consistent with reports that elicitor-induced expression of squalene epoxidase, cycloartenol synthase, and pathway cytochrome P450s persists for days after the elicitor signal has resolved.^57-59^

## 4.2. Calcium occupies an upstream regulatory position

Four lines of evidence place Ca^2+^ at the head of the cascade. It responded first, at 5 minutes. Its transient was fully resolved before any other variable peaked. LaCl_3_ suppressed the peak by 71% and, more tellingly, suppressed everything downstream of it: 53.7% of the NADPH oxidase response, 49.5% of the H_2_O_2_ response, 43.7% of the NO response, 39.1% of the jasmonate response, and 48.0% of the withanolide gain. No downstream inhibitor affected the Ca^2+^ transient itself: DPI, cPTIO, and DIECA left it within 7% of the chitosan-only value. The mechanistic basis is well established. RBOH proteins carry two EF-hand motifs that bind Ca^2+^ directly and are additionally activated by Ca^2+^-dependent protein kinase phosphorylation of their *N*-terminal region; therefore, cytosolic Ca^2+^ gates the oxidase both allosterically and by phosphorylation.^38^ ^,^ ^39^ That LaCl_3_ removed only approximately half of the oxidative burst while DPI removed three-quarters is consistent with LaCl_3_ acting on plasma membrane channels only and leaving intracellular Ca^2+^ release intact, and with a fraction of apoplastic H_2_O_2_ originating from cell wall peroxidases rather than from RBOH.^35^ ^,^ ^37^ Two caveats should be stated plainly. LaCl_3_ is a lanthanide that blocks several cation channels and can interact with the cell wall; therefore, its effects are not exclusively attributable to a single channel class.^66^ And Fluo-4 in an intact leaf reports a relative, not an absolute, cytosolic Ca^2+^ concentration.^67^ The conclusion that Ca^2+^ is upstream rests not on the pharmacology alone but on the joint evidence of timing, one-directional inhibitor sensitivity, and the structural equation model.

The inhibitor experiments provide strong but qualified causal evidence for the proposed Ca^2+^→ROS→NO→jasmonate signaling pathway. Their principal strength is the asymmetric perturbation pattern across independently targeted nodes: LaCl_3_ reduced all later responses, DPI strongly reduced ROS and the later NO/JA responses without suppressing the initial Ca^2+^ transient, cPTIO markedly removed NO and attenuated JA while largely sparing the ROS peak, and DIECA strongly suppressed JA/JA-Ile while leaving the earlier signals almost intact. This forward-propagating and largely non-reciprocal pattern is difficult to explain by temporal coincidence. However, pharmacological inhibitors are not absolutely pathway-specific, the magnitude of inhibition is incomplete for several steps, and no rescue with exogenous downstream signals or genetic loss-of-function material is included. Therefore, we interpret the data as strong evidence for functional ordering and partial causal dependence rather than definitive proof of an exclusive linear molecular cascade.

### 4.3. ROS act as a signal rather than simply causing oxidative damage

The distinction between redox signaling and oxidative injury is quantitative, and the data allow it to be drawn quantitatively. H_2_O_2_ levels increased by 2.7-fold and returned to baseline within 48 h, whereas malondialdehyde levels increased by 11% and electrolyte leakage by 0.8 percentage points, both transiently. Photosynthesis, *F*_v_/*F*_m_, relative water content, chlorophyll, and shoot biomass were unchanged. Meanwhile, GSH/GSSG and AsA/DHA levels increased, indicating that the pools became more reduced, which is the opposite of what oxidative injury produces.^42^ ^,^ ^43^ The dose‒response experiment strengthens this interpretation by showing where the boundary lies. At 200 and 400 mg L^−1^, membrane injury and biomass loss appeared, and withanolide content decreased; thus, the same elicitor became a stressor once the redox excursion exceeded the buffering capacity of the antioxidant system.^29-31^ The peak of antioxidant enzyme activity at 6 hours, three hours after the H_2_O_2_ maximum, is exactly the lag expected of a system that detects and then corrects a redox excursion, and the sustained 15%–25% elevation through day 7 suggests that elicitation resets antioxidant capacity to a higher steady state rather than merely mopping up a single burst.^41^ The practical implication for medicinal-plant production is that the useful window for chitosan in this species is narrow and can be located empirically: the correct dose is the one that maximizes the integrated H_2_O_2_ signal without moving malondialdehyde or electrolyte leakage, which in our conditions was 100 mg L^−1^.

### 4.4. NO provides a regulatory bridge between redox and hormone signaling

NO occupied an intermediate position in both time (peak at 6 hours) and NO dependency. Its accumulation required upstream events, and DPI and LaCl_3_ suppressed it by 54.1% and 43.7%, respectively. This accumulation was required for a substantial part of the jasmonate response, since cPTIO removed 45.6% of the JA peak. Neither of these effects can be explained by direct chemical interference; therefore, NO is genuinely embedded between the redox and hormonal layers.^44-46^ The effect of NO on the duration rather than the amplitude of the oxidative signal is a more subtle finding. cPTIO barely changed the H_2_O_2_ peak (10.9% suppression) but accelerated its decay and reduced the 0–12 hours integrated signal from 121.6 to 115.8. This is consistent with the *S*-nitrosylation of RBOHD at Cys890, which inhibits superoxide production, and with NO-dependent modulation of catalase and ascorbate peroxidase activity; the net effect of removing NO is a burst of normal height but shorter tail.^47-49^ Because the integrated signal, not the peak, was the better predictor of withanolide content in our correlation analysis, a change in duration of this kind is functionally meaningful even when the peak is unchanged. NO scavenging also has compound-specific consequences. cPTIO suppressed withaferin A accumulation by 37.7% but withanoside V by 40.5%, whereas the upstream blockers suppressed withanoside V much more strongly than withaferin A. Individual branches of withanolide metabolism therefore do not read the NO signal identically, which argues against treating “total withanolides” as the meaningful response variable.^68^ ^,^ ^69^

### 4.5. Jasmonate translates early signals into metabolic outcomes

Jasmonate behaved as the terminal signaling node and as the strongest single predictor of the metabolic outcome. DIECA removed 83.4% of the JA response and 81.5% of the JA-Ile response while leaving Ca2^+^, ROS, and NO nearly intact, confirming that it acted as intended; the resulting 36.1% reduction in the withanolide gain is therefore attributable to the loss of jasmonate rather than to a disturbance of the upstream cascade. In the structural equation model, jasmonate carried the largest direct path coefficient to day-7 withanolides (*β* = 0.369) of any signaling variable. The enrichment of *α*-linolenic acid metabolism in the untargeted data provides independent, non-hormonal support for this conclusion, since that pathway is the biosynthetic route to jasmonate and its enrichment was detected without any prior assumption about which metabolites to look for.^51^ ^,^ ^54^ Two limits on interpretation should be respected. First, DIECA inhibits lipoxygenase and can also act as a metal chelator and antioxidant; therefore, a residual off-target contribution cannot be excluded.^70^ Second, and more importantly, our data show that jasmonate is *required* for the full withanolide response and *predicts* it, but they do not demonstrate direct transcriptional regulation of withanolide biosynthetic genes by the JA-Ile-COI1-JAZ-MYC module in this species. Establishing that would require expression analysis of pathway genes, JAZ degradation assays, and ideally genetic manipulation, none of which was performed here. The literature showing that exogenous methyl jasmonate upregulates squalene epoxidase, sterol methyltransferase, and pathway P450s in *W. somnifera* makes such regulation plausible, but plausibility is not demonstration.^57-59^ ^,^ ^71^ The comparatively large jasmonate coefficient should therefore be interpreted as evidence that jasmonate is the strongest direct statistical predictor of day-7 withanolides within the prespecified SEM, not as proof that this path is uniquely or exclusively causal. Its mechanistic interpretation gains support only because the same direction is independently consistent with signal timing and DIECA-mediated attenuation of the withanolide response. Therefore, the evidence is sufficient to support jasmonate as an important mediator of subsequent withanolide accumulation, but not as the sole obligatory driver. This conclusion rests on convergence of temporal precedence, selective pharmacological attenuation, strong JA/JA-Ile AUC–withanolide associations, and the largest direct SEM path from jasmonate to day-7 withanolides. Because DIECA is not completely pathway-specific and no JA rescue, COI1/JAZ perturbation, or withanolide-pathway gene-expression experiment was performed, we have deliberately limited the causal claim to a jasmonate-dependent contribution to the full elicitor response.

### 4.6. Withanolide biosynthesis is regulated as a metabolic network

The differential responses of individual compounds are best explained by network behavior rather than uniform pathway activation. Withaferin A increased by 113.5%, while the withanosides increased by approximately 63%, shifting the composition of the pool toward the highly oxygenated aglycone. The ranking of inhibitor sensitivities differed between compounds, with DPI being the most effective against withaferin A and LaCl3 being the most effective against withanoside V. If a single upstream switch controls the entire pathway, these rankings are identical. The metabolomic data indicated that the precursor supply, not merely terminal enzyme activity, was upregulated: cholesterol, campesterol, and 24-methylenecholesterol increased by 20%–26%, and the aggregate precursor pool increased by 61.7%, alongside a 41% increase in the number of detectable withanolide-type features. If chitosan had simply activated the last steps of the pathway, precursors would have been depleted rather than enriched.^6^ ^,^ ^7^ ^,^ ^27^ The apparent absence of a growth-defense trade-off is worth noting because it is often assumed rather than measured. Shoot dry weight fell by only 3.9% and was not significantly different, and photosynthesis was unchanged, while the withanolide pool grew by 84%. At the dose used, the carbon and reducing equivalents redirected into steroidal-lactone synthesis were evidently a small enough fraction of the plant’s budget to be absorbed without a measurable growth penalty–consistent with the view that trade-offs become detectable mainly when defense investment is sustained or when resources are already limiting.^28^ ^,^ ^72^ Whether the same holds under field water or nutrient limitation is untested here. This interpretation also reflects annotation confidence: the quantified target withanolides provide Level 1 anchors for the pathway, whereas many additional untargeted steroidal-lactone and precursor features remain Level 2 or Level 3. Their coordinated behavior strengthens the pathway-level inference, but individual identities should remain regarded as putative until confirmed against authentic standards or by orthogonal structural methods such as NMR.

These abundance changes should not be equated with direct flux measurements. The simultaneous accumulation of cholesterol, campesterol, 24-methylenecholesterol, the aggregate precursor pool, and numerous withanolide products supports coordinated activation of precursor supply and specialized metabolism; however, a larger metabolite pool can arise from changes in production, utilization, compartmentation, transport, or degradation. Therefore, we interpret the data as supporting increased metabolic capacity or pathway engagement, while treating increased flux as a testable hypothesis. Stable-isotope tracing and quantitative metabolic-flux analysis will be required to determine whether carbon flow through the sterol-to-withanolide pathway actually increased and to quantify its magnitude.

### 4.7. Signal dynamics are more informative than single-time measurements

The kinetic parameters extracted here–time to peak, peak amplitude, duration, time to return to baseline, and area under the curve–performed more analytical work than any single concentration measurement. Areas under the curve predicted day-7 withanolide content with *r* = 0.88–0.92 and consistently outperformed peak amplitudes, showing that a signal’s information content includes how long it lasts, not only how high it rises. This is the operational form of the “calcium signature” concept, extended to ROS, NO, and jasmonate.^73-75^ The time-lagged analysis made the point most directly. Ca^2+^predicted the eventual withanolide phenotype strongly at 15–60 minutes and not at all from 3 hours onward; H_2_O_2_ became predictive at 30 minutes and remained predictive to 24 hours; NO and jasmonate switched on later. A study that sampled only at 24 hours would have found a good NO and jasmonate correlation, no Ca^2+^ correlation, and would have concluded that Ca^2+^ is irrelevant to withanolide accumulation–a conclusion the inhibitor data show to be incorrect. The sampling schedule, in other words, determines which mechanism a study is capable of discovering.^36^ ^,^ ^62^ We suggest that reporting the four kinetic descriptors used here, rather than single-time concentrations, should become routine in elicitation studies on medicinal plants. It costs additional sampling points but converts a descriptive comparison into a testable causal model.

### 4.8. Pharmaceutical relevance of elicitor-responsive metabolites

Selecting docking ligands from the compounds that increased, rather than from the literature, links the agronomic and pharmacological halves of this study. Chitosan enriched withaferin A most strongly in both absolute and relative terms, and withaferin A also gave the best predicted binding score against IκB kinase *β* (−9.8 kcal mol^−1^, marginally better than the redocked reference ligand), the most favorable MM-GBSA energy (−40.2 kcal mol-1), and the most stable 100 ns trajectory. The convergence is encouraging: the treatment preferentially produces the compound that the computational assessment prioritizes. The choice of IKKβ as the single target class was made on biological grounds, since inhibition of NF-κB activation is the most consistently reported molecular action of withaferin A, and the 3.4 Å proximity of the ring-A enone to Cys99 in our top pose is compatible with the covalent mechanism reported for this compound.^8^ ^,^ ^9^ ^,^ ^76^ That said, docking as performed here models non-covalent binding only, so the pose should be read as a plausible pre-covalent orientation rather than as a model of the covalent adduct.

The predicted ADMET profiles add a practical dimension: the aglycones were predicted to be well absorbed and Lipinski-compliant, whereas the glycosylated withanosides were not, which is consistent with the pharmacokinetic literature and means that an elicitation strategy that preferentially enriches aglycones may be more useful than one that raises total withanolide content indiscriminately.^14^ ^,^ ^15^ ^,^ ^77^ These statements are predictions of interaction potential and physicochemical behavior. Docking scores, MM-GBSA energies, and in silico ADMET values are not measures of efficacy, potency, or safety, and the well-documented weak correlation between docking scores and experimental affinity means that they should be used only to prioritize compounds for experimental testing.^78^ Enzyme-inhibition assays, cellular target-engagement experiments, and pharmacokinetic measurements remain necessary before any therapeutic claims can be made.

## 5. Conclusion

Chitosan induces a temporally coordinated signaling cascade in *W. somnifera,* that begins with a transient cytosolic Ca^2+^ elevation peaking at 15 minutes, proceeds through an NADPH oxidase-dependent oxidative burst at 3 hours and an NO signal at 6 hours, and culminates in jasmonate accumulation at 12 hours. Pharmacological disruption established which nodes are necessary for which outcomes: blocking Ca^2+^ influx suppressed every downstream event, inhibiting NADPH oxidase removed most of the oxidative burst and much of the NO and jasmonate response, scavenging NO shortened the oxidative signal and reduced jasmonate, and inhibiting jasmonate biosynthesis affected only the hormonal step. None of the blockades abolished withanolide accumulation, indicating parallel and partially redundant routes from perception to biosynthesis. Total withanolides increased by 84% within seven days, with a disproportionate gain in withaferin A, and untargeted metabolomics showed that this reflected broad reprogramming of steroid, terpenoid-backbone, and *α*-linolenic acid metabolism together with a 61.7% enlargement of the withanolide precursor pool—all achieved without measurable membrane injury, photosynthetic loss, or biomass penalty. Integrated early signals predicted the day-7 metabolic phenotype with correlations of 0.88–0.92, and a structural equation model explained 98.4% of the variance in total withanolide content with jasmonate as the strongest direct predictor. Computational assessment prioritized withaferin A and withanolide A as the elicitor-responsive metabolites most worth taking into experimental pharmacological validation. Signal timing, and not signal magnitude alone, is what predicts elicitor-driven specialized metabolism in this species. The pharmacological data should be read as strong directional and functional support for this ordering, not as definitive molecular proof: incomplete inhibition, possible off-target actions, and the absence of rescue or genetic validation leave room for parallel and feedback pathways. The SEM component should consequently be regarded as supportive rather than causal evidence: its high explanatory power and significant paths demonstrate consistency of the data with the proposed network, whereas causal confidence derives from convergence with temporal ordering and pharmacological perturbation and remains provisional pending genetic, rescue, and other orthogonal validation. The metabolomics component should therefore be interpreted hierarchically: Level 1 target compounds provide high-confidence chemical anchors, while Level 2 and Level 3 features contribute supportive, progressively lower-confidence evidence for pathway enrichment and broader metabolic reprogramming rather than definitive compound-specific identification.

Therefore, metabolomic evidence demonstrates coordinated changes in metabolite abundance and pathway enrichment, not direct metabolic flux. The 61.7% expansion of the measured precursor pool, together with the accumulation of multiple withanolide end products, supports enhanced pathway engagement; however, confirmation of increased carbon flux will require isotope-labeled precursor tracing or other dedicated flux-analysis approaches. Future targeted validation of the most influential Level 2/3 features with authentic standards, retention-time confirmation, and, where necessary, NMR would further strengthen the chemical basis of the pathway assignment. These results support jasmonate as a major, but not exclusive, mediator linking the early Ca^2+^-ROS-NO cascade to subsequent withanolide accumulation. The conclusion is supported by temporal precedence, strong JA/JA-Ile-withanolide associations, and the 36.1% attenuation of the chitosan-induced withanolide gain when jasmonate biosynthesis was inhibited; however, direct JA-Ile-COI1-JAZ-MYC control of withanolide-pathway genes remains to be experimentally demonstrated.

## Data Availability

The data generated or analyzed during the current study are available from the corresponding author upon reasonable request.

## Statement on guidelines

All experimental studies and experimental materials involved in this research are in full compliance with relevant institutional, national and international guidelines and legislation.

## Abbreviations

APX: ascorbate peroxidase
APX: ascorbate peroxidase
AsA: ascorbate
AUC: area under the curve
CAT: catalase
cPTIO: 2-(4-carboxyphenyl)-4,4,5,5-tetramethylimidazoline-1-oxyl-3-oxide
DAF-FM DA: 4-amino-5-methylamino-2′,7′-difluorofluorescein diacetate
DAS: days after sowing
DHA: dehydroascorbate
DIECA: sodium diethyldithiocarbamate
DPI: diphenyleneiodonium
DW: dry weight
EL: electrolyte leakage
FDR: false discovery rate
FW: fresh weight
GR: glutathione reductase
GSH: reduced glutathione
GSSG: oxidized glutathione
JA: jasmonic acid
JA-Ile: jasmonoyl-isoleucine
MDA: malondialdehyde
MD: molecular dynamics
NO: nitric oxide
PPFD: photosynthetic photon flux density
QC: quality control
RBOH: respiratory burst oxidase homolog
RFU: relative fluorescence units
ROS: reactive oxygen species
RWC: relative water content
SEM: structural equation model
SOD: superoxide dismutase
